# Systematic review of *Aedes aegypti* control trials suggests publication bias related to author disclosure of conflicts of interest

**DOI:** 10.1371/journal.pntd.0013914

**Published:** 2026-01-14

**Authors:** Abdisalam A. Abdi, Jose G. Juarez, Trevor Harris, Tereza Magalhaes, Gabriel L. Hamer

**Affiliations:** 1 Department of Entomology, Texas A&M University, College Station, Texas, United States of America; 2 Sustainable Sciences Institute, Managua, Nicaragua; 3 Current address: College of Veterinary Medicine & Biomedical Sciences, Texas A&M University, College Station, Texas, United States of America; 4 Department of Statistics, Texas A&M University, College Station, Texas, United States of America; University of Heidelberg, GERMANY

## Abstract

**Background:**

*Aedes aegypti* mosquitoes transmit multiple arboviruses, including dengue, Zika, chikungunya, and yellow fever, resulting in a large global disease burden. Vector control remains the key strategy to prevent transmission due to the absence of widely available vaccines or treatments. Many studies evaluate control approaches, yet only a subset are published in peer-reviewed journals. One potential contributor to selective reporting, or publication bias, could be a conflict of interest (COI), defined as employment by a for-profit company conducting the trial, or a financial interest tied to the tool’s intellectual property.

**Methodology/principal findings:**

We conducted a systematic literature review of *Ae. aegypti* control trials from 2010 to 2022 to test the hypothesis that published trials with author-declared COI report a higher average level of *Ae. aegypti* suppression than publications whose authors declare no COI. Inclusion criteria required entomological outcomes (adult abundance or immature indices) with baseline and post-intervention data for both treated and untreated areas. Studies limited to laboratory, semi-field, or virus-only outcomes were excluded. We identified 51 publications that met the inclusion criteria. The studies with declared COI reported a 56.7% reduction in *Ae. aegypti* population, significantly higher than the 34.5% reduction in studies declaring no COI. The 51 studies were published in 26 different journals and eight (30.7%) did not have standard publishing policies that include the reporting of authors’ COI statements in the published articles.

**Conclusions/significance:**

Our findings suggest that author-reported COI is associated with higher mosquito population suppression. This association may reflect the use of more effective interventions in COI-affiliated studies or publication bias. We also observed inconsistencies in COI policies and the display of COI statements across journals, underscoring the need for standardized and transparent reporting.

## Author summary

Many studies evaluate *Aedes aegypti* mosquito vector control approaches, but only a subset are published. One possible contributor to selective reporting, or publication bias, is the presence of a conflict of interest (COI), such as employment by the company conducting the trial or a financial conflict tied to intellectual property. We conducted a systematic review of field trials of *Ae. aegypti* control from 2010-2022 and examined whether studies in which authors disclose a COI report higher vector suppression than studies declaring no COI. Among 51 studies that met inclusion criteria, those with declared COI reported greater *Ae. aegypti* reduction of 56.7% compared to 34.5% from studies that declared no COI. We also observed that COI statements are not consistently published across journals. These findings suggest potential publication bias and underscore the need for consistent, transparent COI disclosure policies to strengthen credibility and interpretation of vector control evidence.

## Introduction

Once restricted to Africa, the *Aedes aegypti* (Linnaeus, 1762) mosquito is now widespread across tropical and sub-tropical regions of the world due to human-mediated transport and trade, ecological adaptation to urban container habitats [1], and human feeding behavior [2,3]. *Aedes aegypti* has become the primary vector of agents of diseases such as dengue, Zika, yellow fever, and chikungunya viruses [4–6], which places an estimated 5.7 billion people at risk of exposure to *Aedes*-borne viruses in suitable areas [7,8]. The lack of approved vaccines or treatments for these *Aedes*-borne viruses makes vector control strategies the key factor to curtail disease transmission. Studies have assessed the substantial economic burden of major *Aedes*-borne diseases globally and the costs associated with vector surveillance and control programs targeting this mosquito [9–12].

The global health challenge posed by *Ae. aegypti* results in large control campaigns implemented by public health agencies to reduce vector populations. While area-wide control programs by governmental agencies have observed success [13–16], the burden of disease remains high which requires researchers to evaluate existing control tools or develop innovative methods to suppress *Ae. aegypti* populations [17–19]. A significant challenge in advancing the field is the underreporting of field trials that evaluate vector control tools but yield negative or null results, such research regularly fails to get published [20]. This lack of publication results in publication bias which arises from the selective publication of studies based on the direction and magnitude of their findings; studies without statistical significance (i.e., negative results) are less likely to be published [21–25]. Another source of publication bias is that studies are often conducted by companies or owners of intellectual property with direct interests in the field. This context could create a conflict of interest (COI) when determining which studies are submitted to journals for publication and which are not. While it is difficult to systematically evaluate the lack of *Ae. aegypti* control studies with negative data in peer-reviewed literature, it is possible to evaluate the potential for COI to influence which studies get published. Our hypothesis is that authors having a COI will report a higher average level of *Ae. aegypti* suppression achieved by the control activity compared to publications with authors not having a COI. To test this hypothesis, we conducted a systematic literature review of all *Ae. aegypti* control trials from 2010 to 2022 to investigate the level of reported population suppression as it relates to the authors disclosure of a COI.

## Methods

### Search strategy and selection criteria

We conducted a systematic literature review using Web of Science (WoS) and PubMed. This review used keyword searches for “*Aedes aegypti* control” and “*Aedes aegypti* intervention” from 2010 through 2022. We used 2010–2022 to capture the modern era of *Aedes aegypti* control trials spanning the pre- and post-Zika virus emergence and the more recent journal adoption of COI-disclosure policies, improving comparability of reporting across studies. When searching in WoS and PubMed, we selected the “all fields” option, which searches across all searchable fields in a single query. That allowed us to easily find our search terms in any field. The review identified studies that conducted control trials evaluating the efficacy of vector control tools against *Ae. aegypti*.

All publications resulting from the keyword search were judged based on the following inclusion and exclusion criteria. We included primary studies reporting results of an intervention or control trial on *Ae. aegypti* field populations, studies in which the intervention outcome variable reflects free-ranging *Ae. aegypti* populations, studies that included an entomological outcome variable of mosquitos - adult female *Ae. aegypti* abundance or any immature indices. Additionally, the study needed to have mosquito surveillance data in intervention areas receiving control and reference areas not receiving control - nearby control (e.g., neighboring house) did not count as an adequate control and the study needed to have both pre-intervention (baseline) and post-intervention data presented for both intervention and control communities/clusters.

We excluded insectary or semi-field trials, studies focused on virus as an outcome (e.g., only epidemiological outcome variables or mosquito infection with virus as an outcome variable); studies involving larvicide interventions where only treated larval habitat and control larval habitat are sampled, and review articles. The systematic review was conducted by a single author (AAA) and all candidate papers meeting inclusion criteria were discussed by two authors (AAA and GLH) to arrive at the final determination of exclusion or inclusion. Studies meeting inclusion criteria were categorized based on the authors, their affiliations, and the conflict of interest (COI) sections of the published articles. This categorization was used to determine the presence or absence of COI. Studies were classified as Declared COI, Declared no COI, or No statement (no COI/competing-interests text displayed). When no COI statement appeared in the article, we classified the study as “Declared no COI” to give the authors the benefit of the doubt, recognizing that some journals may omit or fail to display disclosures even when submitted. This classification was determined from the article’s COI text, and funding statements. We did not adjust author declarations using affiliations or funding; mentions of company employment are cited only in the Results/Discussion to illustrate variability in reporting relative to International Committee of Medical Journal Editors (ICMJE) guidance [26], and were not used to reassign COI status.

For transparency, we present analyses both ways: (i) a combined analysis treating “No statement” papers as “Declared no COI,” and (ii) a restricted analysis including only studies that explicitly declared no COI (excluding “No statement”).

### Data extraction

Not all studies reported a standardized result from intervention trials. Therefore, we used the Henderson formula [27–30], to calculate the percentage of *Ae. aegypti* population suppression for each study. See (S1 Text) for an example calculation; extraction details are provided in (S1 Data).

### Henderson formula

We estimated the percentage of *Aedes aegypti* population suppression using the following formula:


Percentage of control=100-[(T/U)×100]

T = Treatment area

U = Untreated area (control)

Where, T is equal to the posttreatment mean divided by the pretreatment mean in the treatment (intervention) area, and

U is the posttreatment mean divided by the pretreatment mean in the control area.

This allowed us to extract the level of efficacy achieved by the intervention by measuring the percent reduction in immature indices (e.g., larval index, pupae per house, pupal index) and adult female *Ae. aegypti*. Using the level of *Ae. aegypti* population suppression from each study, we present the means (± standard error, SE) for studies that declared COIs and those that declared no COIs.

The studies included in this review were geographically diverse, with interventions conducted across regions, some heavily affected by *Ae. aegypti* borne diseases and others not.

### Statistical analysis

We calculated the mean percentage reduction of the *Ae. aegypti* population across studies. Data normality was tested using the Shapiro-Wilk test and homogeneity of variances using Levene’s test. Due to non-normal distribution (Shapiro-Wilk test, p < 0.05), we used the Wilcoxon rank-sum test to compare reduction percentages between groups with COI and without declared COI.

We also used quantile regression to measure the potential impact of COIs on the percent reduction of *Ae. aegypti* in each study, with COI as the main predictor and adjusting for intervention type (Insecticidal, Suppression, Community, Replacement) and geographic region. Countries were grouped into macro-regions (Africa, Americas, Asia, Oceania) to maintain adequate statistical power while accounting for geographic variation. Quantile regression allows us to study the impact of COIs at all levels of the *Ae. aegypti* reduction distribution, i.e., we can separately determine whether a COI are more influential when population suppression is high or low (see S1 Text). Complete statistical analysis code is available in S2 Text. Furthermore, unlike standard linear regression, quantile regression does not require conditional normality for inference and is robust to outliers. This makes quantile regression an appropriate choice for our data because it is heavily skewed and contains many outliers due to highly negative values based on the Henderson formula, which suggests higher vector abundance in the intervention sites compared to the control sites. The results of this analysis provide insights into the relationship between COI and reported mosquito population suppression.

All analyses and figures were conducted using R (version 4.5.1; R Foundation for Statistical Computing, Vienna) [31]. The map was generated with the sf and ggplot2 packages. Country borders were taken from Natural Earth “Admin 0 – Countries” (1:50m, public domain) via rnaturalearth. Borders were joined to study counts by ISO3 codes produced with countrycode. Antarctica was excluded; coordinates are WGS 84 (EPSG:4326) [32–36].

## Results

**Fig 1:**
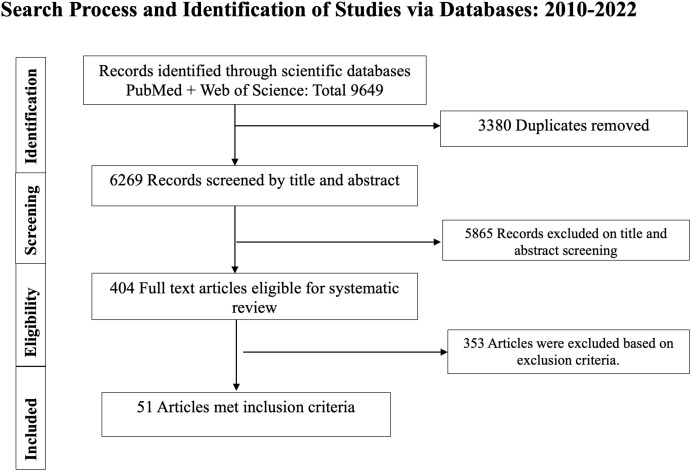
Flowchart for the systematic review to identify eligible studies of *Ae. aegypti* control trials from 2010 - 2022.

The keyword search in PubMed and Web of Science for years 2010–2022 resulted in 9649 total papers, of which 3380 were duplicates (Fig 1). Of the 6269 papers screened, 5865 were excluded based on titles and abstracts. Of the 404 papers read in more detail, 353 were excluded based on inclusion and exclusion criteria. Therefore, we identified 51 publications from 2010-2022 that met the inclusion criteria for this systematic review of *Ae. aegypti* control trials (Table 1).

**Table 1:** Conflict-of-interest (COI) reporting for 51 *Aedes aegypti* studies evaluating intervention tools to achieve population control published across 26 journals (see S1 Text, Table A for journal abbreviations).

Publications	Journal Abbreviation	Journal publishes COI on every article*	COI statement displayed in this article†	Authors declared a COI in this article#
Vazquez-Prokopec et al., 2022 [37]	Sci Rep	Yes	Yes	No
Martín-Park et al., 2022 [38]	PLoS Negl Trop Dis	Yes	Yes	No
Williams et al., 2022 [39]	SN Appl Sci	Yes	Yes	No
Forsyth et al., 2022 [40]	PLoS Negl Trop Dis	Yes	Yes	No
Lenhart et al., 2022 [41]	PLoS Negl Trop Dis	Yes	Yes	No
Manrique-Saide et al., 2021 [42]	Trop Med Int Health	No	No	No
Beebe et al., 2021 [43]	Proc Natl Acad Sci U S A	Yes	Yes	Yes
de Castro Poncio et al., 2021 [44]	J Infect Dis	Yes	Yes	Yes
Manrique-Saide et al., 2021 [45]	PLoS Negl Trop Dis	Yes	Yes	No
Juarez et al., 2021 [46]	J Appl Ecol	Yes	Yes	No
Harris et al., 2021 [47]	Pest Manag Sci	Yes	Yes	No
Gato et al., 2021 [48]	Insects	Yes	Yes	No
Devine et al., 2021 [49]	PLoS Negl Trop Dis	Yes	Yes	No
Holston et al., 2021 [50]	Am J Trop Med Hyg	No	No	No
Hustedt et al., 2021 [51]	Am J Trop Med Hyg	No	No	No
Gopalan et al., 2021 [52]	Pathog. Glob. Health	No	Yes	No
Pinto et al., 2020 [53]	Mem Inst Oswaldo Cruz	No	No	No
Hamid et al., 2020 [54]	Bull Entomol Res	Yes	Yes	Yes
Newton-Sánchez et al., 2020 [55]	Int J Public Health	Yes	Yes	No
Crawford et al., 2020 [56]	Nat Biotechnol	Yes	Yes	Yes
Gunathilaka et al. 2020 [57]	Parasit Vectors	Yes	Yes	No
Ahmad Zaki et al., 2020 [58]	Trop Med. Infect Dis	Yes	Yes	No
Bonnet et al., 2020 [59]	Infect Dis Poverty	Yes	Yes	No
Bohari et al., 2020 [60]	PLoS One	Yes	Yes	Yes
Garcia et al., 2020 [61]	Parasit Vectors	Yes	Yes	No
Lenhart et al., 2020 [62]	PLoS Negl Trop Dis	Yes	Yes	No
Brelsfoard et al., 2019 [63]	Insects	Yes	Yes	No
Kittayapong et al., 2019 [64]	PLoS Negl Trop Dis	Yes	Yes	No
Barrera et al., 2018 [65]	Parasit Vectors	Yes	Yes	No
Oo et al., 2018 [66]	Parasit Vectors	Yes	Yes	Yes
Ponlawat et al., 2017 [67]	J Am Mosq Control Assoc	No	No	No
Abad-Franch et al., 2017 [68]	PLoS Med	Yes	Yes	No
Garziera et al., 2017 [69]	Entomol Exp Appl	No	No	No
Toledo et al., 2017 [70]	PLoS Negl Trop Dis	Yes	Yes	No
Nagpal et al., 2016 [71]	PLoS One	Yes	Yes	No
Setha et al., 2016 [72]	PLoS Negl Trop Dis	Yes	Yes	Yes
Carvalho et al., 2015 [73]	PLoS Negl Trop Dis	Yes	Yes	Yes
Toledo et al., 2015 [74]	PLoS One	Yes	Yes	No
Quintero et al., 2015 [75]	Trans R Soc Trop Med Hyg	Yes	Yes	No
Caprara et al., 2015 [76]	Trans R Soc Trop Med Hyg	Yes	Yes	No
Mitchell-Foster et al., 2015 [77]	Trans R Soc Trop Med Hyg	Yes	Yes	No
Barrera et al., 2014 [78]	J Med Entomol	No	No	No
Tsunoda et al., 2013 [79]	Parasit Vectors	Yes	Yes	No
Lenhart et al., 2013 [80]	Am J Trop Med Hyg	No	Yes	No
Castro et al., 2012 [81]	Trans R Soc Trop Med Hyg	Yes	Yes	No
Arunachalam et al., 2012 [82]	Pathog Glob Health	No	No	No
Martínez-Ibarra et al., 2012 [83]	J Vector Ecol	No	No	No
Abeyewickreme et al., 2012 [84]	Pathog Glob Health	No	No	No
Kittayapong et al., 2012 [85]	Pathog Glob Health	No	No	No
Rizzo et al., 2012 [86]	BMC Public Health	Yes	Yes	No
Marcombe et al., 2011 [87]	PLoS Negl Trop Dis	Yes	Yes	No

**Abbreviation:** COI, conflict of interest.

* **Journal-level practice.** Yes = COI/Competing Interests statements are displayed on all articles the journal publishes; “No” = not displayed on all articles (includes sporadic or never).

*†* **Article-level display for this study.** Yes = this article’s PDF/HTML includes a COI/Competing Interests statement; No = no COI statement is displayed.

*#* **Article-level author declaration.** Yes = authors explicitly declared a COI in this article*;* No = authors did not declare or no COI*.*

### Geographic distribution of studies

**Fig 2:**
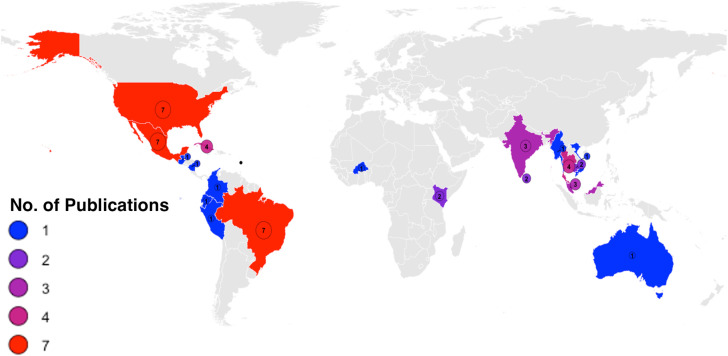
Geographic distribution of studies included in the systematic review of *Aedes aegypti* control trials (2010–2022). Countries are shaded by the number of included publications; circles show the count at each country centroid with both circle size and color scale with count. Basemap: Natural Earth Admin 0 – Countries (1:50m), public domain, accessed via the rnaturalearth R package (https://www.naturalearthdata.com/about/terms-of-use/). Coordinates: WGS 84 (EPSG:4326). No proprietary map tiles were used.

Fig 2 illustrates the geographic distribution of the studies included in this systematic review, highlighting the countries where *Ae. aegypti* control efforts were conducted between 2010 and 2022. From these studies, we extracted data on the journal’s conflict of interest reporting policies, whether authors disclosed any potential conflicts (Tables 1 and A in S1 Text) and the level of mosquito population suppression achieved in the intervention areas (Table B in S1 Text).

**Fig 3:**
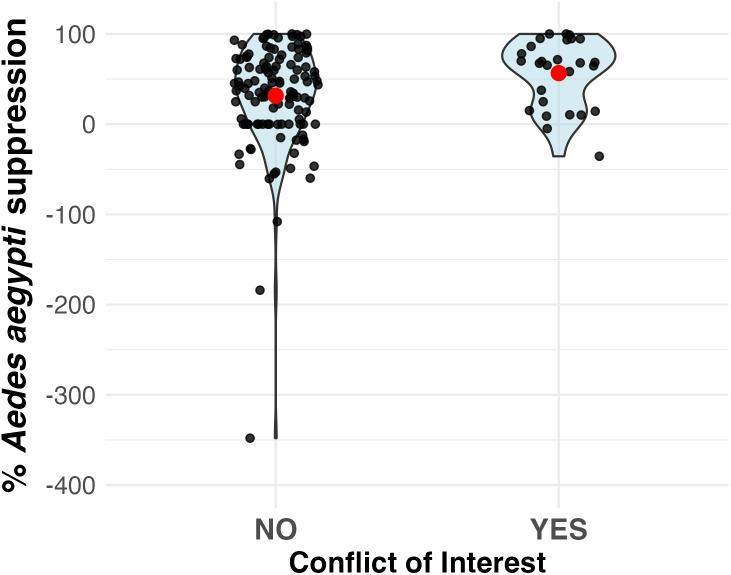
The percentage suppression in *Aedes aegypti* population in relation to conflict of interest (COI). The studies categorized as ‘No’ for COI include studies where authors either declared no COI or nothing about COI was reported.

For studies where authors either declared no COI or No statement of COI was reported, the average *Ae. aegypti* population suppression was 34.5% (SE = 4.2; 95% CI 26.2–42.7), compared to 56.7% (SE = 7.0; 95% CI 42.9–70.4) suppression in studies where authors reported the presence of COI (p-value = 0.025; Fig 3). The mean difference (declared COI declared no COI) was 22.2 percentage points (95% CI 6.1–38.3). After adjusting for intervention type and geographic region using median quantile regression, the COI effect remained significant (β = 0.322, SE = 0.125, p = 0.011). Relative to insecticidal interventions, community-based strategies were associated with significantly higher mosquito reduction (β = 0.308, p < 0.001), while replacement and suppression strategies showed no detectable differences. *Aedes aegypti* population suppression was significantly higher in the Americas (β = 0.448, p < 0.001) and Asia (β = 0.324, p = 0.003) compared to Africa, with no difference observed for Oceania. The presence of COI was independently associated with *Ae. aegypti* population suppression across all regions and intervention types.

Because not all journals consistently publish COI statements, we performed a restricted analysis limited to studies that explicitly declared no COI. This showed mean *Ae. aegypti* population suppression of 26.5% for studies declaring no COI compared to 56.7% in studies with declared COI (Wilcoxon p = 0.007). The adjusted model showed an even stronger COI effect (β = 0.397, SE = 0.165, p = 0.018), with the Americas showing significantly higher *Ae. aegypti* suppression than Africa (β = 0.330, p = 0.0006). These findings confirm the robustness of the COI association across different analytical approaches.

**Fig 4:**
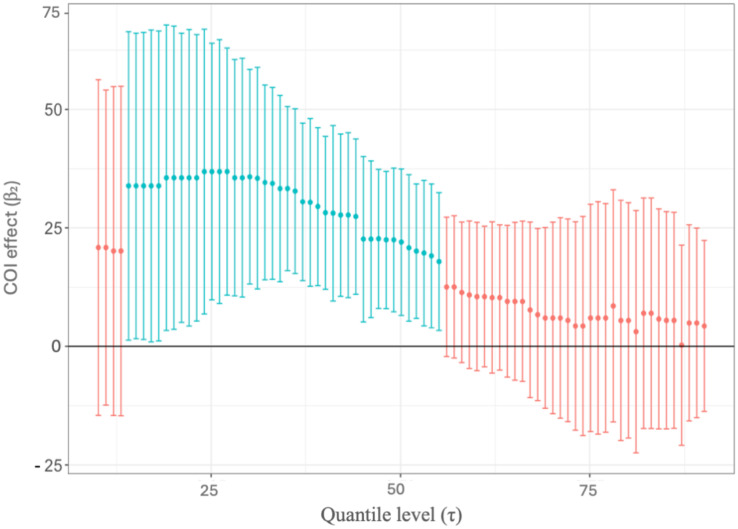
Estimated conflict of interest (COI) coefficients (β₂) across different quantile levels (τ) with ±2 standard errors, showing how the effect of COI varies across the distribution of *Ae. aegypti* population suppression rates. The x-axis shows quantile levels (τ) from 0 to 100, which correspond to the rate of *Aedes aegypti* population suppression reported by the study. The y-axis displays the magnitude of the COI effect (β₂), which refers to the strength of the coefficient. Vertical lines represent ±2 standard errors around each estimate. Blue points and error bars indicate statistically significant coefficients (p < 0.05). Red points and error bars represent non-significant coefficients.

To further understand the impact of COI on study outcomes, we analyzed the distribution of COI effects across varying levels of suppression efficacy using a quantile regression adjusted for intervention type and macro-region. Of all the studies reporting the presence of COI, 39.3% fall below the *Ae. aegypti* population suppression value of 60%, while 60.7% are above. Of all the studies that either reported no COI or nothing about COI was reported, 65.0% fall below the *Ae. aegypti* population suppression value of 60%, while 35.0% are above. When suppression efficacy is high (τ>60there is no significant difference between studies that report a COI and studies that report no COI. The association between COI status and suppression was strongest (more positive) at lower levels of the distribution (τ<60), meaning that studies with lower reported population suppression of *Ae. aegypti* have a greater difference with those studies that declared COI and those that declared no COI (Fig 4). These results identify an association, where a larger COI effect was observed for studies reporting minimal evidence of *Ae. aegypti* population suppression. This suggests that COI may disproportionately influence studies with lower reported *Ae. aegypti* suppression efficacy. In these adjusted quantile models, the COI effect remained generally positive and of similar magnitude, suggesting that intervention type and geographic region did not fully explain the higher suppression observed in COI studies.

The 51 papers that met the inclusion criteria for this study were published in 26 different peer-reviewed journals. An unexpected result was that eight out of these 26 journals (30.7%) did not have standard publishing policies that include the reporting of authors’ COI statements in the published articles. While some of these journals sporadically disclose COI in a subset of published articles, other journals do not report COI for any published article.

Finally, published articles by different author teams had inconsistencies in how authors interpreted which circumstances represented a conflict requiring disclosure. For example, according to the International Committee of Medical Journal Editors (ICMJE) definition of COI [26,88], authors affiliated with either for-profit or not-for-profit organizations should be disclosed. As an example, Brelsfoard et al. [63] reported no COI, but according to the ICMJE guidelines, the authors should have disclosed that multiple authors were employees of the for-profit company that conducted the trial.

## Discussion

This systematic review of *Ae. aegypti* population control trials suggests that publication bias may exist in the peer-reviewed literature. While this study is unable to detect the presence of publication bias due to studies that have undesirable outcomes (e.g., negative results showing a lack of suppression of *Ae. aegypti* populations), our results indicate that authors of publications with a COI tend to report a significantly higher levels of *Ae. aegypti* population suppression compared to authors of publications with no COI. Two or more mechanisms could result in this observation. One is that investigators of research trials evaluating mosquito control approaches may be using more effective tools when they have some type of COI present. If a patent exists around a vector control tool, that implies sufficient rigor and novelty which would suggest the vector control tool works to control *Ae. aegypti* at the population level. Accordingly, the observed differences in suppression could partly reflect differences in intervention effectiveness rather than bias introduced by COI itself. Another possibility is that investigators with a COI are conducting multiple vector control trials and only the trials with successful population suppression of *Ae. aegypti* are being submitted to journals for publication. This mechanism may be occurring, given this study’s observation that studies reporting lower levels of *Ae. aegypti* population suppression below 60% were more likely to be reported among author teams with no declared COI (either explicitly ‘no COI’ or no statement). Our quantile regression analysis indicates that the COI suppression association is most pronounced at lower efficacy levels (τ < 60) suggesting fewer COI-declared studies in the lower-suppression range. Notably, when suppression efficacy is high (τ > 60), there is no significant difference between studies reporting a COI and those with no COI, suggesting that any potential publication bias may be concentrated among studies with lower effectiveness. This suggests that author teams with a COI are less likely to publish an intervention trial when the level of success was low. These findings indicate an association between the reporting of COI in vector control trials and the level of *Ae. aegypti* population suppression, which does not imply direct causation.

Similar patterns have been observed in pharmaceutical research, where multiple systematic reviews have consistently demonstrated that industry-sponsored studies tend to report more favorable outcomes [25,89–92]. The implication that not all studies are equally likely to be published, particularly those with negative results, highlights the presence of publication bias in the mosquito vector control literature. This bias skews the available evidence which can lead to wasted resources as programs use tools that are less effective than evidence would suggest and results in redundant research. The underreporting of negative or null results can lead to unnecessary duplication of ineffective interventions, hindering progress in developing truly effective vector control solutions [93].

Several limitations of this study should be considered while interpreting these results. We only searched two literature databases (PubMed and Web of Science), so relevant studies indexed elsewhere, in the grey literature, or in non-English journals would have likely been missed. Not including papers published in non-English likely reduced the geographic distribution of studies observed in Fig 2. Initial screening was performed by a single author, with candidate papers meeting inclusion criteria being discussed with a second author. Literature screening independently by two authors, and then comparing any discrepancies in the resulting lists, would have reduced the risk of missing papers. COI status was determined strictly from each article’s published disclosures, author affiliations, and funding notes; when no disclosure appeared in the publication, we classified the study as “declared no COI.” Because journal policies and the reporting of disclosure statements vary, this unexpected observation confounded the testing of our hypothesis comparing the reporting of COI and the success of *Ae. aegypti* control trials. Finally, while we used the Henderson formula to standardize suppression values across studies, this approach does not remove ecological or operational heterogeneity (e.g., seasonality, sampling effort, or intervention intensity).

Another important discovery by this study is the wide heterogeneity in policies regarding COI among the entomological and public health journals that publish results of vector control trials. While most journals require authors to disclose COI during manuscript submission on their online portals, eight out of the 26 journals (30.7%) which included the papers in this study meeting inclusion criteria did not disclose COI in the publication. Some publishers of journals, such as PLOS, report author’s competing interests as a mandatory section in all publications. Other journals, such as the Journal of the American Mosquito Control Association, do not report COI in any of their published articles, which is concerning. Among the nine journals published by the Entomological Society of America, the reporting of COI has been sporadic and only appears in a small fraction of the papers in these journals. Our team recently published a study on a vector control tool in the Journal of Medical Entomology [94] and during the page proofs stage of the publication, we made a request to the editors and production staff that we include a COI statement, which they agreed to. We explained our rationale for why COI is important to report in all publications and the Director of Publications, Communications, and Marketing for the Entomological Society of America brought this subject to the attention of the Publication Council, and they are recommending that all future papers of ESA journals include a COI section.

Beyond the concern that some journals do not include a mandatory COI section in all publications is the variation in the definition of what type of COI authors should disclose. Our cursory review of different journal policies revealed variation in the author guidelines as it relates to COI. Organizations such as the International Committee of Medical Journal Editors, the World Association of Medical Editors, and the Committee on Publication Ethics (COPE) have launched initiatives to establish international standards for COI disclosures. COPE requires its 7000 member journals to comply with its Code of Conduct for Journal Editors [95], but some journals still do not adhere to these guidelines. This study’s results show that some authors are not reporting a COI which could be due to a journal not requiring that section in the published version of the paper, or because the authors are not clear on the definition of a COI. Indeed, the ICMJE definition of COI is not straight-forward, and describes any scenario where professional judgement concerning the publication of results could be influenced by a secondary interest [88]. Many journals have strict policies regarding human subjects research, reviewed and approved by Institutional Review Boards, and animal subjects research, reviewed by Institutional Animal Care and Use Committees. Journal editorial staff ensure that publications involving human subjects or animal subjects have proper permits indicating research compliance have been met. Additionally, anonymous peer reviewers of manuscripts submitted to journals are asked to comment on research compliance as it relates to human and animal subjects. However, it appears journals do not police the topic of COI with the same rigor. For example, simply having the author’s affiliations as a company conducting the control trial meets the definition of COI according to the ICMJE [88], and studies meeting inclusion criteria in this systematic review did not disclose those COIs [96]. Increased oversight from journals and institutions could help the scientific community improve transparency, accountability and reduce undisclosed COI [95,97]. To enhance transparency and address these issues, we recommend that journals adopt a standardized COI definition ideally based on internationally recognized guidelines, such as those provided by COPE and the ICMJE, and communicate this clearly in the author guidelines. This would ensure consistency and help authors understand exactly what needs to be disclosed. Importantly, the presence of a COI does not, by itself, invalidate scientific findings; however, consistent and transparent disclosure is essential for evaluating credibility and interpreting results in context. In addition, journal editors could strengthen oversight related to COI in a manner analogous to existing practice for human and animals subjects research.

In conclusion, the integrity of vector control research is paramount for advancing effective interventions against *Ae. aegypti* and other important arthropod vectors of human disease. This systematic literature review suggests that authors that disclose a conflict of interest tend to report a higher level of *Ae. aegypti* population suppression compared to authors that with declared no COI. This association of COI was most significant for studies reporting a lower level of *Ae. aegypti* population suppression, which is consistent with authors with a COI being less likely to publish studies with lower success. These results suggest potential publication bias in scientific literature related to the control of one of the world’s most important mosquito vectors of human pathogens. This study also indicates that journals are not consistent in the guidelines of COI and the practice of reporting COI in all publications. We emphasize a call for action is needed for journals, authors, and institutions to increase transparency, oversight, and accountability to reduce potential publication bias in the peer-reviewed literature and allow the field of organized vector control to make efficient advancements in controlling vectors responsible for a large burden of human disease.

## Supporting information

S1 Text A worked example of the Henderson formula used to estimate mosquito population suppression, along with an explanation of the quantile regression approach and the modeling strategy used to assess the impact of conflict of interest (COI) on reported outcomes. Includes Table A (journal/COI reporting summary) and Table B (extracted suppression outcomes).
(DOCX)

S2 Text R code for quantile regression analysis of conflict of interest effects with macro-region adjustment. (PDF)

S1 PRISMA 2020 Checklist Completed PRISMA 2020 checklist for the systematic review. PRISMA 2020 checklist is available under CC BY 4.0; citation guidance: https://www.prisma-statement.org/citing-prisma-2020.
(PDF)

S1 Data Detailed Henderson-Tilton calculations and source data for all 226 comparisons from 51 *Aedes aegypti* control studies. Includes three sheets: (1) Main calculation - detailed extraction with pre/post intervention data, (2) MAIN - complete dataset for main analysis, (3) RESTRICTED - dataset limited to studies with explicit COI declarations. Note: Some values were extracted from figures when not reported numerically in text.
(XLSX)
