# Supplementary material for: Systematic review of Aedes aegypti control trials suggests publication bias related to author disclosure of conflicts of interest
Source: PLoS Negl Trop Dis. 2026 Jan 14;20(1):e0013914. doi: 10.1371/journal.pntd.0013914 (PMC12829951; doi:10.1371/journal.pntd.0013914)
Supplement: S1 Text — Includes Table A (journal/COI reporting summary) and Table B (extracted suppression outcomes). (DOCX) [file pntd.0013914.s001.docx]

**S1 Text.** A worked example of the Henderson formula used to estimate mosquito population suppression, along with an explanation of the quantile regression approach and modeling strategy used to assess the impact of conflict of interest (COI) on reported outcomes.

**Henderson formula**

We estimated the percentage of *Aedes aegypti* population suppression using the following formula:

Percentage of control = 100 - [(T/U) × 100]

T = Treatment area

U = Untreated area (control)

Where, T is equal the posttreatment mean divided by the pretreatment mean in the treatment (intervention) area, and

U is the posttreatment mean divided by the pretreatment mean in the control area.

Example article [1]

House index.

Untreated(control):

Pretreatment = 0.8383.

Posttreatment = 3.1664

Treatment area:

Pretreatment = 1.2944.

Posttreatment = 2.0497

Untreated = $\frac{\mathrm{Posttreatment}}{\mathrm{Pretreatment}}$ = $\frac{3.1664}{0.8383}$ = 3.7771

Treatment = $\frac{\mathrm{Posttreatment}}{\mathrm{Pretreatment}}= \frac{2.0497}{1.2944}$ = 1.583

Percentage of control = 100 – [($\frac{T}{U}$ $\times100)]=100-\left[ \left( \frac{1.583}{3.7771} \right)\times100 \right]=100-41.9=58.1\%$

Reduction = 58.1%

**Quantile regression**

Quantile regression is a regression technique for modeling the conditional quantiles of the response variable as a function of the independent variables. That is, given a quantile level $\tau\in(0, 1)$ we model the $\tau$’th quantile of $Y$, denoted $Q(Y, \tau)$ as:

$$Q\left( Y, \tau\right)= X\beta$$

Where $X$is our design matrix including an intercept column.

We considered a range of quantiles $\tau=10, 11, \ldots,89, 90$ to measure the impact of COI on each level of the bulk of the distribution. We excluded any external quantiles $\tau<10$and $\tau>90$ because there are very few data points in these ranges, which leads to quantile regressions well known for instability in estimating extreme conditional quantiles [2]. For all quantiles in the bulk range, we model the conditional quantile of the response (percent reduction) as a linear combination of COI and intervention type.

Our model is:

$$Q\left( Y, \tau\right)= \beta_{0}+X_{1}\beta_{1}+X_{2}\beta_{2}$$

Where $X_{1}$ is a categorical variable containing the intervention type, grouped into 10 categories (Appendix Table 2), and $X_{2}$ is an indicator variable of whether a COI was present or not. Our primary interest is on $\beta_{2}$, however, we need to control for intervention type due to potential confounding between the response and the intervention type (i.e. some intervention types may be naturally more effective than others). We fit the above model for each quantile level and use the classic quantile regression bootstrapping procedure [3] to estimate the sampling distribution of each beta coefficient.

**Table A in S1 Text.** Full journal names, abbreviations, COI reporting fields, and study site for the 51 included studies**.**

Study site indicates the country where the field trial was conducted (city/region noted when relevant). Journal abbreviations match those used in Table 1

| **Publications** | **Journal name** | **Journal Abbreviation** | **Journal publishes COI on every article*** | **COI statement displayed in this article†** | **Authors declared a COI in this article#** | **Study Site** |
| --- | --- | --- | --- | --- | --- | --- |
| Vazquez-Prokopec et al., 2022[4] | SCIENTIFIC REPORTS | Sci Rep | YES | YES | NO | Mexico |
| Martín-Park et al., 2022[5] | PLOS NEGLECTED TROPICAL DISEASES | PLoS Negl Trop Dis | YES | YES | NO | Mexico |
| Williams et al., 2022[6] | SN APPLIED SCIENCES | SN Appl Sci | YES | YES | NO | USA |
| Forsyth et al., 2022[7] | PLOS NEGLECTED TROPICAL DISEASES | PLoS Negl Trop Dis | YES | YES | NO | Kenya |
| Lenhart et al., 2022[8] | PLOS NEGLECTED TROPICAL DISEASES | PLoS Negl Trop Dis | YES | YES | NO | Venezuela |
| Manrique-Saide et al., 2021[9] | TROPICAL MEDICINE & INTERNATIONAL HEALTH | Trop Med Int Health | NO | NO | NO | Mexico |
| Beebe et al., 2021[10] | PROCEEDINGS OF THE NATIONAL ACADEMY OF SCIENCES OF THE UNITED STATES OF AMERICA | Proc Natl Acad Sci U S A | YES | YES | YES | Australia |
| de Castro Poncio et al., 2021[11] | JOURNAL OF INFECTIOUS DISEASES | J Infect Dis | YES | YES | YES | Brazil |
| Manrique-Saide et al., 2021[12] | PLOS NEGLECTED TROPICAL DISEASES | PLoS Negl Trop Dis | YES | YES | NO | Mexico |
| Juarez et al., 2021[13] | JOURNAL OF APPLIED ECOLOGY | J Appl Ecol | YES | YES | NO | USA |
| Harris et al., 2021[14] | PEST MANAGEMENT SCIENCE | Pest Manag Sci | YES | YES | NO | USA |
| Gato et al., 2021[15] | INSECTS | Insects | YES | YES | NO | Cuba |
| Devine et al., 2021[16] | PLOS NEGLECTED TROPICAL DISEASES | PLoS Negl Trop Dis | YES | YES | NO | Mexico |
| Holston et al., 2021[17] | AMERICAN JOURNAL OF TROPICAL MEDICINE AND HYGIENE | Am J Trop Med Hyg | NO | NO | NO | Nicaragua |
| Hustedt et al., 2021[18] | AMERICAN JOURNAL OF TROPICAL MEDICINE AND HYGIENE | Am J Trop Med Hyg | NO | NO | NO | Cambodia |
| Gopalan et al., 2021[19] | PATHOGENS AND GLOBAL HEALTH | Pathog. Glob. Health | NO | YES | NO | India |
| Pinto et al., 2020[20] | MEMORIAS DO INSTITUTO OSWALDO CRUZ | Mem Inst Oswaldo Cruz | NO | NO | NO | Brazil |
| Hamid et al., 2020[21] | BULLETIN OF ENTOMOLOGICAL RESEARCH | Bull Entomol Res | YES | YES | YES | Malaysia |
| Newton-Sánchez et al., 2020[22] | INTERNATIONAL JOURNAL OF PUBLIC HEALTH | Int. J. Public Health | YES | YES | NO | Mexico |
| Crawford et al., 2020[23] | NATURE BIOTECHNOLOGY | Nat. Biotechnol | YES | YES | YES | USA |
| Gunathilaka et al. 2020[24] | PARASITES & VECTORS | Parasit Vectors | YES | YES | NO | Sri Lanka |
| Ahmad Zaki et al., 2020[25] | TROPICAL MEDICINE AND INFECTIOUS DISEASE | Trop. Med. Infect. Dis. | YES | YES | NO | Malaysia. |
| Bonnet et al., 2020[26] | INFECTIOUS DISEASES OF POVERTY | Infect Dis Poverty | YES | YES | NO | Burkina Faso |
| Bohari et al., 2020[27] | PLOS ONE | PLoS One | YES | YES | YES | Malaysia |
| Garcia et al., 2020[28] | PARASITES & VECTORS | Parasit Vectors | YES | YES | NO | Brazil. |
| Lenhart et al., 2020[29] | PLOS NEGLECTED TROPICAL DISEASES | PLoS Negl Trop Dis | YES | YES | NO | Peru |
| Brelsfoard etal., 2019[30] | INSECTS | Insects | YES | YES | NO | USA |
| Kittayapong et al., 2019[31] | PLOS NEGLECTED TROPICAL DISEASES | PLoS Negl Trop Dis | YES | YES | NO | Thailand |
| Barrera et al., 2018[32] | PARASITES & VECTORS | Parasit Vectors | YES | YES | NO | USA |
| Oo et al., 2018[33] | PARASITES & VECTORS | Parasit Vectors | YES | YES | YES | Myanmar |
| Ponlawat et al., 2017[34] | JOURNAL OF THE AMERICAN MOSQUITO CONTROL ASSOCIATION | J Am Mosq Control Assoc | NO | NO | NO | Thailand |
| Abad-Franch et al., 2017[35] | PLOS MEDICINE | PLoS Med | YES | YES | NO | Brazil |
| Garziera et al., 2017[36] | ENTOMOLOGIA EXPERIMENTALIS ET APPLICATA | Entomol Exp Appl | NO | NO | NO | Brazil |
| Toledo et al., 2017[37] | PLOS NEGLECTED TROPICAL DISEASES | PLoS Negl Trop Dis | YES | YES | NO | Cuba |
| Nagpal et al., 2016[38] | PLOS ONE | PLoS One | YES | YES | NO | India |
| Setha et al., 2016[39] | PLOS NEGLECTED TROPICAL DISEASES | PLoS Negl Trop Dis | YES | YES | YES | Cambodia |
| Carvalho et al., 2015[40] | PLOS NEGLECTED TROPICAL DISEASES | PLoS Negl Trop Dis | YES | YES | YES | Brazil |
| Toledo et al., 2015[41] | PLOS ONE | PLoS One | YES | YES | NO | Cuba |
| Quintero et al., 2015[42] | TRANSACTIONS OF THE ROYAL SOCIETY OF TROPICAL MEDICINE AND HYGIENE | Trans R Soc Trop Med Hyg | YES | YES | NO | Colombia |
| Caprara et al., 2015[1] | TRANSACTIONS OF THE ROYAL SOCIETY OF TROPICAL MEDICINE AND HYGIENE | Trans R Soc Trop Med Hyg | YES | YES | NO | Brazil |
| Mitchell-Foster et al., 2015[43] | TRANSACTIONS OF THE ROYAL SOCIETY OF TROPICAL MEDICINE AND HYGIENE | Trans R Soc Trop Med Hyg | YES | YES | NO | Ecuador |
| Barrera et al., 2014[44] | JOURNAL OF MEDICAL ENTOMOLOGY | J Med Entomol | NO | NO | NO | USA |
| Tsunoda et al., 2013[45] | PARASITES & VECTORS | Parasit Vectors | YES | YES | NO | Vietnam |
| Lenhart et al.2013[46] | AMERICAN JOURNAL OF TROPICAL MEDICINE AND HYGIENE | Am J Trop Med Hyg | NO | YES | NO | Thailand |
| Castro et al., 2012[47] | TRANSACTIONS OF THE ROYAL SOCIETY OF TROPICAL MEDICINE AND HYGIENE | Trans R Soc Trop Med Hyg | YES | YES | NO | Cuba |
| Arunachalam et al., 2012[48] | PATHOGENS AND GLOBAL HEALTH | Pathog Glob Health | NO | NO | NO | India |
| Martínez-Ibarra et al., 2012[49] | JOURNAL OF VECTOR ECOLOGY | J Vector Ecol | NO | NO | NO | Mexico |
| Abeyewickreme et al., 2012[50] | PATHOGENS AND GLOBAL HEALTH | Pathog Glob Health | NO | NO | NO | Sri Lanka |
| Kittayapong et al., 2012[51] | PATHOGENS AND GLOBAL HEALTH | Pathog Glob Health | NO | NO | NO | Thailand |
| Rizzo et al., 2012[52] | BMC PUBLIC HEALTH | BMC Public Health | YES | YES | NO | Guatemala |
| Marcombe et al.,2011[53] | PLOS NEGLECTED TROPICAL DISEASES | PLoS Negl Trop Dis | YES | YES | NO | Island of Martinique, Caribbean |
|  |  |  |  |  |  |  |

*** Journal-level practice.** “Yes” = COI/Competing Interests statements are displayed on all articles the journal publishes; “No” = not displayed on all articles (includes sporadic or never)

**† Article-level display for this study.** “Yes” = the PDF/HTML of this article includes a COI/Competing Interests statement; “No” = no COI statement is displayed

**# Article-level author declaration.** “Yes” = authors explicitly declared a COI in this article; “No” = authors did not declare a COI (includes explicit “no COI” or no declaration).

**Abbreviation: COI, conflict of interest.**

**Table B in S1 Text.** Summary of data extracted from studies evaluating *Aedes aegypti* population suppression methods. The table details the percentage reduction in mosquito populations and related entomological outcomes for various interventions. Complete Henderson calculations for all 226 comparisons with source data are provided in S1 Data. For studies where exact numerical values were not reported in text, data were extracted from figures using digital estimation; all extraction details are documented in S1 Data.

| **Author** | **Intervention  Type** | **% Reduction *Ae. aegypti***^†^ | **Multiple entomological outcome variables^#^** | **Nested treatment variable*** |
| --- | --- | --- | --- | --- |
| Vazquez-Prokopec et al., 2022 [4] | Insecticidal | 53.19% | Adult female *Ae.aegypti* per house | Actellic 300CS |
| Vazquez-Prokopec et al., 2022 [4] | Insecticidal | 5% | Adult female *Ae.aegypti* per house | SumiShield 50WG |
| Martín-Park et al., 2022 [5] | Suppression | 15.71% | No. outdoor females/ BG trap |  |
| Martín-Park et al., 2022 [5] | Suppression | 89.09% | No. indoor females per house |  |
| Williams et al., 2022 [6] | Insecticidal | 45.43% | Adult female *Aedes* | Females caught in BGS trap |
| Williams et al., 2022 [6] | Insecticidal | 33.91% | Adult female *Aedes* | Female and male *Ae. aegypti* collected from the BG-Sentinel |
| Williams et al., 2022 [6] | Insecticidal | 45.10% | Adult female *Aedes* | Average no. of females collected (Landing Rate Count, LRC) |
| Forsyth et al., 2022 [7] | Community | 47.83% | Container Index |  |
| Forsyth et al., 2022 [7] | Community | 24.81% | House Index |  |
| Lenhart et al., 2022 [8] | Insecticidal | 73.94% | Breteau Index | insecticide treated curtains (ITCs) |
| Lenhart et al., 2022 [8] | Insecticidal | 28.21% | Pupae per Person (PPI), | insecticide treated curtains (ITCs) |
| Lenhart et al., 2022 [8] | Insecticidal | 51.11% | House Index | insecticide treated curtains (ITCs) |
| Lenhart et al., 2022 [8] | Insecticidal | 59.77% | Container Index | insecticide treated curtains (ITCs) |
| Lenhart et al., 2022 [8] | Insecticidal | 67.46% | Breteau Index | insecticide-treated water storage jar covers (ITJCs) |
| Lenhart et al., 2022 [8] | Insecticidal | 47.35% | Pupae per Person (PPI), | insecticide-treated water storage jar covers (ITJCs) |
| Lenhart et al., 2022 [8] | Insecticidal | 60.80% | House Index | insecticide-treated water storage jar covers (ITJCs) |
| Lenhart et al., 2022 [8] | Insecticidal | 62.95% | Container Index | insecticide-treated water storage jar covers (ITJCs) |
| Lenhart et al., 2022 [8] | Insecticidal | 87.24% | Breteau Index | ITC+ITJC |
| Lenhart et al., 2022 [8] | Insecticidal | 97.28% | Pupae per Person (PPI), | ITC+ITJC |
| Lenhart et al., 2022 [8] | Insecticidal | 76.49% | House Index | ITC+ITJC |
| Lenhart et al., 2022 [8] | Insecticidal | 84.57% | Container Index | ITC+ITJC |
| Manrique-Saide et al., 2021[9] | Insecticidal | 28.75% | Houses positive for *Aedes* females |  |
| Manrique-Saide et al., 2021[9] | Insecticidal | 64.56% | Number of female *Aedes* per house |  |
| Beebe et al., 2021 [10] | Replacement | 58.32% | sterile males released | Mourilyan T1 |
| Beebe et al., 2021 [10] | Replacement | 14.31% | Sterile males released | South Johnstone T2 |
| Beebe et al., 2021 [10] | Replacement | 68.64% | Sterile males released | Goondi Bend T3 |
| de Castro Poncio et al., 2021 [11] | Suppression | 69.95% | Sterile males released |  |
| Manrique-Saide et al., 2021 [12] | Suppression | 30.23% | Houses positive for *Aedes* females 6 Month |  |
| Manrique-Saide et al., 2021 [12] | Suppression | 29.41% | Houses positive for *Aedes* females. 12 Months |  |
| Manrique-Saide et al., 2021 [12] | Suppression | 13.40% | No. of female *Aedes* per house. 6 months |  |
| Manrique-Saide et al., 2021 [12] | Suppression | -33.33% | No. of female *Aedes* per house 12 months |  |
| Juarez et al., 2021 [13] | Suppression | -278.18% | Adult female *Aedes* | Low-income year 1 |
| Juarez et al., 2021 [13] | Suppression | 16.67% | Adult female *Aedes* | Low-income year 2 |
| Juarez et al., 2021 [13] | Suppression | 90.91% | Adult female *Aedes* | Middle Income year 1 |
| Juarez et al., 2021 [13] | Suppression | 10.00% | Adult female Aedes | Middle Income year 2 |
| Harris et al., 2021 [14] | Insecticidal | 46.69% | Adult population | SITE 1 |
| Harris et al., 2021 [14] | Insecticidal | 28.35% | Adult population | SITE 2 |
| Gato et al., 2021 [15] | Suppression | 42.09% | Sterile males released |  |
| Devine et al., 2021 [16] | Insecticidal | 44.50% | Female *Ae. aegypti* |  |
| Holston et al., 2021 [17] | Community | 61.16% | House index | Mid-Study 3 month |
| Holston et al., 2021 [17] | Community | 70.63% | House index | Exit 1 YEAR |
| Holston et al., 2021[17] | Community | 84.62% | House index | Post-Study 2 years |
| Holston et al., 2021 [17] | Community | 70.93% | Container index | Mid-Study 3 month |
| Holston et al., 2021 [17] | Community | 84.04% | Container index | Exit 1 YEAR |
| Holston et al., 2021 [17] | Community | 89.61% | Container index | Post-Study 2 years |
| Holston et al., 2021 [17] | Community | 71.17% | Breteau index | Mid-Study 3 month |
| Holston et al., 2021 [17] | Community | 75.13% | Breteau index | Exit 1 YEAR |
| Holston et al., 2021 [17] | Community | 89.04% | Breteau index | Post-Study 2 years |
| Holston et al., 2021 [17] | Community | 57.02% | Pupae per Container | Mid-Study 3 month |
| Holston et al., 2021 [17] | Community | 98.98% | Pupae per Container | Exit 1 YEAR |
| Holston et al., 2021 [17] | Community | 99.44% | Pupae per Container | Post-Study 2 years |
| Holston et al., 2021 [17] | Community | 58.62% | Pupae per household | Mid-Study 3 month |
| Holston et al., 2021 [17] | Community | 99.31% | Pupae per household | Exit 1 YEAR |
| Holston et al., 2021 [17] | Community | 99.47% | Pupae per household | Post-Study 2 years |
| Hustedt et al., 2021 [18] | Insecticidal | 38.89% | Adult female *Aedes* | Guppies. Dry season |
| Hustedt et al., 2021 [18] | Insecticidal | 57.04% | Adult female *Aedes* | Guppies.  Light rain |
| Hustedt et al., 2021 [18] | Insecticidal | -33.33% | Adult female *Aedes* | Guppies. Heavy rain |
| Hustedt et al., 2021 [18] | Insecticidal | 36.36% | Adult female *Aedes* | Guppies + PPF. Dry season |
| Hustedt et al., 2021 [18] | Insecticidal | 57.58% | Adult female *Aedes* | Guppies + PPF. Light rain |
| Hustedt et al., 2021 [18] | Insecticidal | 27.27% | Adult female *Aedes* | Guppies + PPF. Heavy rain |
| Hustedt et al., 2021 [18] | Insecticidal | 62.84% | Breateau index | Guppies. Dry season |
| Hustedt et al., 2021 [18] | Insecticidal | 57.55% | Breateau index | Guppies.  Light rain |
| Hustedt et al., 2021 [18] | Insecticidal | 40.09% | Breateau index | Guppies. Heavy rain |
| Hustedt et al., 2021 [18] | Insecticidal | 44.86% | Breateau index | Guppies + PPF. Dry season |
| Hustedt et al., 2021 [18] | Insecticidal | 48.85% | Breateau index | Guppies + PPF. Light rain |
| Hustedt et al., 2021 [18] | Insecticidal | 30.28% | Breateau index | Guppies + PPF. Heavy rain |
| Hustedt et al., 2021 [18] | Insecticidal | 93.25% | Pupae per person | Guppies. Dry season |
| Hustedt et al., 2021 [18] | Insecticidal | 87.73% | Pupae per person | Guppies.  Light rain |
| Hustedt et al., 2021 [18] | Insecticidal | 80.71% | Pupae per person | Guppies. Heavy rain |
| Hustedt et al., 2021 [18] | Insecticidal | 42.73% | Pupae per person | Guppies + PPF. Dry season |
| Hustedt et al., 2021 [18] | Insecticidal | 77.69% | Pupae per person | Guppies + PPF. Light rain |
| Hustedt et al., 2021 [18] | Insecticidal | 18.18% | Pupae per person | Guppies + PPF. Heavy rain |
| Gopalan et al., 2021 [19] | Community | 62.72% | House index |  |
| Gopalan et al., 2021 [19] | Community | 1.60% | Container index |  |
| Gopalan et al., 2021 [19] | Community | 47.39% | Breteau index |  |
| Pinto et al., 2020 [20] | Insecticidal | 15.97% | Ovitraps Positivity Index (OPI) | Powder |
| Pinto et al., 2020 [20] | Insecticidal | 0.62% | Ovitraps Positivity Index (OPI) | Liquid |
| Pinto et al., 2020 [20] | Insecticidal | 61.99% | Eggs Density Index (EDI) | Powder |
| Pinto et al., 2020 [20] | Insecticidal | 56.24% | Eggs Density Index (EDI) | Liquid |
| Pinto et al., 2020 [20] | Insecticidal | 67.25% | Eggs Average Index (EAI) | Powder |
| Pinto et al., 2020 [20] | Insecticidal | 55.00% | Eggs Average Index (EAI) | Liquid |
| Hamid et al., 2020 [21] | Insecticidal | **10.4%** | Ovitrap index | TORS |
| Hamid et al., 2020 [21] | Insecticidal | **24.9%** | larval index | TORS |
| Hamid et al., 2020 [21] | Insecticidal | 8.9% | Ovitrap index | ADD |
| Hamid et al., 2020 [21] | Insecticidal | 37.6% | larval index | ADD |
| Hamid et al., 2020 [21] | Insecticidal | -4.9% | Ovitrap index | TORS AND ADD |
| Hamid et al., 2020 [21] | Insecticidal | -35.6% | larval index | TORS AND ADD |
| Newton-Sánchez et al., 2020 [22] | Community | 55.01% | Breteau Index |  |
| Crawford et al., 2020 [23] | Replacement | 98.11% | Sterile males released | Treatment and Control 1 |
| Crawford et al., 2020 [23] | Replacement | 92.66% | Sterile males released | Treatment and Control 1 |
| Crawford et al., 2020 [23] | Replacement | 98.84% | Sterile males released | Treatment and Control 1 |
| Gunathilaka et al. 2020 [24] | Insecticidal | 96.49% | Mean larvae per plant | Week 1-5 |
| Gunathilaka et al. 2020 [24] | Insecticidal | 70.12% | Mean larvae per plant | Week 6-7 |
| Gunathilaka et al. 2020 [24] | Insecticidal | 56.19% | Mean larvae per plant | Week 8-11 |
| Gunathilaka et al. 2020 [24] | Insecticidal | 45.18% | Mean larvae per plant | Week 12 |
| Ahmad Zaki et al., 2020 [25] [25] | Insecticidal | 22.40% | Positive Ovitrap Index -  Outdoor | Pressure sprayer |
| Ahmad Zaki et al., 2020 [25] | Insecticidal | 60.43% | Positive Ovitrap Index - Semi-indoor | Pressure sprayer |
| Ahmad Zaki et al., 2020 [25] | Insecticidal | -27.36% | Positive Ovitrap Index -  Outdoor | ULV spray |
| Ahmad Zaki et al., 2020 [25] | Insecticidal | -49.24% | Positive Ovitrap Index - Semi-indoor | ULV spray |
| Ahmad Zaki et al., 2020 [25] | Insecticidal | 29.13% | Positive Ovitrap Index -  Outdoor | Mist blower |
| Ahmad Zaki et al., 2020 [25] | Insecticidal | 30.10% | Positive Ovitrap Index - Semi-indoor | Mist blower |
| Ahmad Zaki et al., 2020 [25] | Insecticidal | 35.19% | mean larvae per ovitrap Outdoor | Pressure sprayer |
| Ahmad Zaki et al., 2020 [25] | Insecticidal | 50.00% | mean larvae per ovitrap Semi-Idoor | Pressure sprayer |
| Ahmad Zaki et al., 2020 [25] | Insecticidal | 22.22% | mean larvae per ovitrap Outdoor | ULV spray |
| Ahmad Zaki et al., 2020 [25] | Insecticidal | -380.00% | mean larvae per ovitrap Semi-Idoor | ULV spray |
| Ahmad Zaki et al., 2020 [25] | Insecticidal | 74.07% | mean larvae per ovitrap Outdoor | Mist blower |
| Ahmad Zaki et al., 2020 [25] | Insecticidal | -46.67% | mean larvae per ovitrap Semi-Idoor | Mist blower |
| Bonnet et al., 2020 [26] | Community | 30.34% | Container Index |  |
| Bonnet et al., 2020 [26] | Community | 35.15% | House index |  |
| Bonnet et al., 2020 [26] | Community | 25.81% | Breteau Index |  |
| Bonnet et al., 2020 [26] | Community | 39.74% | Pupae per person |  |
| Bohari et al., 2020 27] | Insecticidal | 93.60% | *Aedes* index | 12 weeks during treatment |
| Bohari et al., 2020 [27] | Insecticidal | 100.00% | *Aedes* index | 4 weeks post treatment |
| Bohari et al., 20202 [27] | Insecticidal | 86.30% | Larvae density | 12 weeks during treatment |
| Bohari et al., 2020 [27] | Insecticidal | 100.00% | Larvae density | 4 weeks post treatment |
| Bohari et al., 2020 [27] | Insecticidal | 69.62% | Ovitrap index | 12 weeks during treatment |
| Bohari et al., 2020 [27] | Insecticidal | 67.95% | Ovitrap index | 4 weeks post treatment |
| Garcia et al., 2020 [28] | Insecticidal | 64.02% | Adult aspiration |  |
| Garcia et al., 2020 [28] | Insecticidal | -25.12% | ovitraps |  |
| Lenhart et al., 2020 [29] | Insecticidal | 0.56% | Female *Ae. aegypti* collected per house. |  |
| Brelsfoard etal., 2019 [30] | Insecticidal | -0.16% | Sterile males released |  |
| Brelsfoard etal., 2019 [30] | Insecticidal | 57.66% | Sterile males released |  |
| Brelsfoard etal., 2019 [30] | Insecticidal | 88.23% | Sterile males released |  |
| Kittayapong et al., 2019 [31] | Suppression | 88.33% | Female per house |  |
| Barrera et al., 2018 [32] | Suppression | 78.92% | Female *Ae. aegypti* per sentinel AGO | Arcadio |
| Barrera et al., 2018 [32] | Suppression | 19.16% | Female *Ae. aegypti* per sentinel AGO | Santa Ana |
| Oo et al., 2018 [33] | Insecticidal | 64.07% | Percent of infested containers |  |
| Ponlawat et al., 2017 [34] | Insecticidal | 75.65% | Adult female *Aedes* | Trial I: Patriot + ULDNyG |
| Ponlawat et al., 2017 [34] | Insecticidal | -27.66% | Adult female *Aedes* | Trial I: Patriot + ULDNyG |
| Ponlawat et al., 2017 [34] | Insecticidal | -24.44% | Adult female *Aedes* | Trial I: Patriot + 9MNyG |
| Ponlawat et al., 2017 [34] | Insecticidal | -38.78% | Adult female *Aedes* | Trial I: Patriot + 9M |
| Ponlawat et al., 2017 [34] | Insecticidal | 68.00% | Adult female *Aedes* | Trial I: Twister+ULDNyG |
| Ponlawat et al., 2017 [34] | Insecticidal | -124.00% | Adult female *Aedes* | Trial I: Twister+ULD |
| Ponlawat et al., 2017 [34] | Insecticidal | 61.60% | Adult female *Aedes* | Trial I: Twister+9MNyG |
| Ponlawat et al., 2017 [34] | Insecticidal | 39.39% | Adult female *Aedes* | Trial I: Twister+9M |
| Ponlawat et al., 2017 [34] | Insecticidal | 42.61% | Adult female *Aedes* | Trial II: Twister+ULDNyG |
| Ponlawat et al., 2017 [34] | Insecticidal | 17.14% | Adult female *Aedes* | Trial II: Twister+ULD |
| Ponlawat et al., 2017 [34] | Insecticidal | 47.95% | Adult female *Aedes* | Trial II: Twister+9MNyG |
| Ponlawat et al., 2017 [34] | Insecticidal | 22.02% | Adult female *Aedes* | Trial II: Twister+9M |
| Abad-Franch et al., 2017 [35] | Insecticidal | 86.17% | Aedes juveniles per SBS | citywide |
| Abad-Franch et al., 2017 [35] | Insecticidal | 91.44% | Aedes juveniles per SBS | focal dissemantion |
| Garziera et al., 2017 [36] | Suppression | 70.09% | Eggs per trap | Juazeiro |
| Garziera et al., 2017 [36] | Suppression | 72.25% | Eggs per itrap | Jacobina |
| Garziera et al., 2017 [36] | Suppression | 57.14% | Ovitrap index | Juazeiro |
| Garziera et al., 2017 [36] | Suppression | 44.12% | Ovitrap index | Jacobina |
| Toledo et al., 2017 [37] | Insecticidal | 17.42% | House index (HI) | Insecticide-treated curtain (ITC |
| Toledo et al., 2017 [37] | Insecticidal | 0.69% | Pupal index (PI) | Insecticide-treated curtain (ITC |
| Toledo et al., 2017 [37] | Insecticidal | 37.19% | House index (HI) | Residual insecticide treatment (RIT) in 3 MONTHS |
| Toledo et al., 2017 [37] | Insecticidal | 20.13% | House index (HI) | Residual insecticide treatment (RIT) overall |
| Toledo et al., 2017 [37] | Insecticidal | 45.71% | Pupal index (PI) | Residual insecticide treatment (RIT) in 3 MONTHS |
| Toledo et al., 2017 [37] | Insecticidal | 0.65% | Pupal index (PI) | Residual insecticide treatment (RIT) overall |
| Nagpal et al., 2016 [38] | Insecticidal | 99.42% | Container Index (CI) | Non-transmission months |
| Nagpal et al., 2016 [38] | Insecticidal | 99.15% | House Index (HI) | Non-transmission months |
| Nagpal et al., 2016 [38] | Insecticidal | 100.00% | Pupal Index (PI) | Non-transmission months |
| Nagpal et al., 2016 [38] | Insecticidal | 99.72% | Breteau Index (BI) | Non-transmission months |
| Nagpal et al., 2016 [38] | Insecticidal | 95.06% | Container Index (CI) | Transmission months |
| Nagpal et al., 2016 [38] | Insecticidal | 86.02% | House Index (HI) | Transmission months |
| Nagpal et al., 2016 [38] | Insecticidal | 99.85% | Pupal Index (PI) | Transmission months |
| Nagpal et al., 2016 [38] | Insecticidal | 97.38% | Breteau Index (BI) | Transmission months |
| Setha et al., 2016 [39] | Insecticidal | 15.48% | Pupae pos containers | Peani 2005 |
| Setha et al., 2016 [39] | Insecticidal | 10.51% | Pupae pos containers | Peani 2006 |
| Setha et al., 2016 [39] | Insecticidal | 71.51% | Pupae per house | Peani 2005 |
| Setha et al., 2016 [39] | Insecticidal | 65.29% | Pupae per house | Peani 2006 |
| Setha et al., 2016 [39] | Insecticidal | 67.70% | Adult per house | Peani 2005 |
| Setha et al., 2016 [39] | Insecticidal | 59.57% | Adult per house | Peani 2006 |
| Carvalho et al., 2015 [40] | Suppression | 95.22% | Adult population |  |
| Carvalho et al., 2015 [40] | Suppression | 78.26% | Ovitrap index |  |
| Toledo et al., 2015 [41] | Insecticidal | -52.91% | House Index (HI) |  |
| Quintero et al., 2015 [42] | Insecticidal | -54.95% | Pupae per person index (PPI) | First follow-up - 1st intervention |
| Quintero et al., 2015 [42] | Insecticidal | 18.22% | Pupae per person index (PPI) | Second follow-up - 1st intervention |
| Quintero et al., 2015 [42] | Insecticidal | -184.09% | Pupae per person index (PPI) | First follow-up - 2nd intervention |
| Quintero et al., 2015 [42] | Insecticidal | 60.80% | Pupae per person index (PPI) | Second follow-up - 2nd intervention |
| Quintero et al., 2015 [42] | Insecticidal | 31.01% | Breteau index (BI) | First follow-up - 1st intervention |
| Quintero et al., 2015 [42] | Insecticidal | -44.64% | Breteau index (BI) | Second follow-up - 1st intervention |
| Quintero et al., 2015 [42] | Insecticidal | -19.15% | Breteau index (BI) | First follow-up - 2nd intervention |
| Quintero et al., 2015 [42] | Insecticidal | 5.87% | Breteau index (BI) | Second follow-up - 2nd intervention |
| Quintero et al., 2015 [42] | Insecticidal | 31.89% | Container index (CI) | First follow-up - 1st intervention |
| Quintero et al., 2015 [42] | Insecticidal | -12.27% | Container index (CI) | Second follow-up - 1st intervention |
| Quintero et al., 2015 [42] | Insecticidal | -0.26% | Container index (CI) | First follow-up - 2nd intervention |
| Quintero et al., 2015 [42] | Insecticidal | 53.13% | Container index (CI) | Second follow-up - 2nd intervention |
| Quintero et al., 2015 [42] | Insecticidal | 24.87% | Household index | First follow-up - 1st intervention |
| Quintero et al., 2015 [42] | Insecticidal | -60.39% | Household index | Second follow-up - 1st intervention |
| Quintero et al., 2015 [42] | Insecticidal | -14.93% | Household index | First follow-up - 2nd intervention |
| Quintero et al., 2015 [42] | Insecticidal | -16.87% | Household index | Second follow-up - 2nd intervention |
| Caprara et al., 2015 [1] | Community | 58.08% | House Index (HI) |  |
| Caprara et al., 2015 [1] | Community | 71.88% | Container Index (CI) |  |
| Caprara et al., 2015 [1] | Community | 63.30% | Breteau index (BI) |  |
| Caprara et al., 2015 [1] | Community | 75.40% | Pupae per person index (PPI) |  |
| Mitchell-Foster et al., 2015 [43] | Community | 64.66% | Pupa per person index (PPI) | PPI2012 |
| Barrera et al., 2014 [44] | Suppression | 59.18% | female *Ae. aegypti* per sentinel AGO | BG-Sentinel traps |
| Barrera et al., 2014 [44] | Suppression | 54.55% | female *Ae. aegypti* per sentinel AGO | Sentinel AGO traps |
| Tsunoda et al., 2013 [45] | Insecticidal | -105.88% | Container Index |  |
| Tsunoda et al., 2013 [45] | Insecticidal | 40.04% | House Index |  |
| Tsunoda et al., 2013 [45] | Insecticidal | 73.83% | Pupae per container |  |
| Lenhart et al.2013 [46] | Insecticidal | 6.25% | Breteau Index (BI) |  |
| Lenhart et al.2013 [46] | Insecticidal | -20.19% | House Index (HI) |  |
| Lenhart et al.2013 [46] | Insecticidal | -3.70% | Container Index (CI) |  |
| Lenhart et al.2013 [46] | Insecticidal | -18.53% | Pupa per person index (PPI) |  |
| Castro et al., 2012 [47] | Community | 36.98% | Breteau indices (BI) |  |
| Arunachalam et al., 2012 [48] | Community | 99.24% | Pupae per person index |  |
| Arunachalam et al., 2012 [48] | Community | 77.66% | House index |  |
| Arunachalam et al., 2012 [48] | Community | 84.53% | Container index |  |
| Arunachalam et al., 2012 [48] | Community | 85.52% | Breteau index |  |
| Martínez-Ibarra et al., 2012 [49] | Community | 95.03% | Container index | López Mateos, El Pastor |
| Martínez-Ibarra et al., 2012 [49] | Community | 52.09% | House Index | López Mateos, El Pastor |
| Martínez-Ibarra et al., 2012 [49] | Community | 93.44% | Container index | Veinte de Noviembre, San Jose |
| Martínez-Ibarra et al., 2012 [49] | Community | 51.37% | House Index | Veinte de Noviembre, San Jose |
| Abeyewickreme et al., 2012 [50] | Community | 84.69% | Pupae per 100 persons Index | Month 2 follow-up |
| Kittayapong et al., 2012 [51] | Insecticidal | -8.52% | House Index (HI) | Month 2 follow-up |
| Kittayapong et al., 2012 [51] | Insecticidal | -5.70% | Container Index (CI) | Month 2 follow-up |
| Kittayapong et al., 2012 [51] | Insecticidal | -5.06% | Breteau index (BI) | Month 2 follow-up |
| Kittayapong et al., 2012 [51] | Insecticidal | -0.54% | House Index (HI) | Month 4 follow-up |
| Kittayapong et al., 2012 [51] | Insecticidal | -39.07% | Container Index (CI) | Month 4 follow-up |
| Kittayapong et al., 2012 [51] | Insecticidal | -34.83% | Breteau index (BI) | Month 4 follow-up |
| Kittayapong et al., 2012 [51] | Insecticidal | 13.06% | House Index (HI) | Month 6 follow-up |
| Kittayapong et al., 2012 [51] | Insecticidal | 31.95% | Container Index (CI) | Month 6 follow-up |
| Kittayapong et al., 2012 [51] | Insecticidal | -9.55% | Breteau index (BI) | Month 6 follow-up |
| Rizzo et al., 2012 [52] | Insecticidal | 43.59% | Total Pupae per cluser | 1st intervention |
| Rizzo et al., 2012 [52] | Insecticidal | 81.19% | Totoal Pupae per cluster | 2nd intervention |
| Marcombe et al.,2011 [53] | Insecticidal | -52.80% | Females per trap outside | Pyrethrins |
| Marcombe et al.,2011 [53] | Insecticidal | -16.98% | Females per trap inside | Pyrethrins |
| Marcombe et al.,2011 [53] | Insecticidal | -32.24% | Females per trap outside | Deltamethrin |
| Marcombe et al.,2011 [53] | Insecticidal | 36.28% | Females per trap inside | Deltamethrin |
| Marcombe et al.,2011 [53] | Insecticidal | -27.35% | Weighted Breteau Index | Pyrethrins |
| Marcombe et al.,2011 [53] | Insecticidal | -1.02% | Weighted Breteau Index | Deltamethrin |

† **Data extraction:** Percentage reduction values were calculated using the Henderson formula (see S1 Text) from pre- and post-intervention mosquito abundance data. When exact numerical values were not reported in the manuscript text or tables, data were extracted from published figures using digital estimation tools. While every effort was made to ensure accuracy, extracted values may have minor discrepancies from original data. Complete extraction details, including source data for all calculations, are provided in S1 Data.

**# Multiple entomological outcome variables:** Some studies reported multiple outcome measures (e.g., adult mosquito abundance, larval indices, pupal indices). In these cases, each outcome is listed as a separate comparison in S1 Data, and the most relevant measure for population suppression was used in the primary analysis.

* **Nested treatment variables** describe the particular formulations, tools, or variations of the interventions used in the studies. These variations are often tested within the same study to compare their efficacy or to isolate the effect of specific components within a treatment**.**

**References**

1. Caprara A, Lima JWDO, Peixoto ACR, Motta CMV, Nobre JMS, Sommerfeld J, et al. Entomological impact and social participation in dengue control: a cluster randomized trial in Fortaleza, Brazil. Trans R Soc Trop Med Hyg. 2015;109: 99–105. doi:10.1093/trstmh/tru187

2. Chernozhukov V, Fernández-Val I, Kaji T. Extremal quantile regression. Handbook of Quantile Regression. 2017; 333–362.

3. Hahn J. Bootstrapping Quantile Regression Estimators. Econometric Theory. 1995;11: 105–121.

4. Vazquez-Prokopec GM, Che-Mendoza A, Kirstein OD, Bibiano-Marin W, González-Olvera G, Medina-Barreiro A, et al. Preventive residual insecticide applications successfully controlled *Aedes aegypti* in Yucatan, Mexico. Sci Rep. 2022;12: 21998. doi:10.1038/s41598-022-26577-1

5. Martín-Park A, Che-Mendoza A, Contreras-Perera Y, Pérez-Carrillo S, Puerta-Guardo H, Villegas-Chim J, et al. Pilot trial using mass field-releases of sterile males produced with the incompatible and sterile insect techniques as part of integrated *Aedes aegypti* control in Mexico. PLOS Neglected Tropical Diseases. 2022;16: e0010324. doi:10.1371/journal.pntd.0010324

6. Williams KF, Ramirez S, Lesser CR. Field evaluation of WALS truck-mounted A1 super duty mist sprayer with VectoBac WDG against *Aedes aegypti* (Diptera:Culicidae) populations in Manatee County, Florida. SN Applied Sciences. 2022;4: 50. doi:10.1007/s42452-021-04893-x

7. Forsyth JE, Kempinsky A, Pitchik HO, Alberts CJ, Mutuku FM, Kibe L, et al. Larval source reduction with a purpose: Designing and evaluating a household- and school-based intervention in coastal Kenya. PLOS Neglected Tropical Diseases. 2022;16: e0010199. doi:10.1371/journal.pntd.0010199

8. Lenhart A, Castillo CE, Villegas E, Alexander N, Vanlerberghe V, Stuyft P van der, et al. Evaluation of insecticide treated window curtains and water container covers for dengue vector control in a large-scale cluster-randomized trial in Venezuela. PLOS Neglected Tropical Diseases. 2022;16: e0010135. doi:10.1371/journal.pntd.0010135

9. Manrique‐Saide P, Herrera‐Bojórquez J, Villegas‐Chim J, Puerta‐Guardo H, Ayora‐Talavera G, Parra‐Cardeña M, et al. Protective effect of house screening against indoor *Aedes aegypti* in Mérida, Mexico: A cluster randomised controlled trial. Trop Med Int Health. 2021;26: 1677–1688. doi:10.1111/tmi.13680

10. Beebe NW, Pagendam D, Trewin BJ, Boomer A, Bradford M, Ford A, et al. Releasing incompatible males drives strong suppression across populations of wild and *Wolbachia*-carrying *Aedes aegypti* in Australia. Proc Natl Acad Sci U S A. 2021;118: e2106828118. doi:10.1073/pnas.2106828118

11. de Castro Poncio L, Dos Anjos FA, de Oliveira DA, Rebechi D, de Oliveira RN, Chitolina RF, et al. Novel sterile insect technology program results in suppression of a field mosquito population and subsequently to reduced Incidence of dengue. J Infect Dis. 2021;224: 1005–1014. doi:10.1093/infdis/jiab049

12. Manrique-Saide P, Herrera-Bojórquez J, Medina-Barreiro A, Trujillo-Peña E, Villegas-Chim J, Valadez-González N, et al. Insecticide-treated house screening protects against Zika-infected *Aedes aegypti* in Merida, Mexico. PLoS Negl Trop Dis. 2021;15: e0009005. doi:10.1371/journal.pntd.0009005

13. Juarez JG, Chaves LF, Garcia‐Luna SM, Martin E, Badillo‐Vargas I, Medeiros MCI, et al. Variable coverage in an Autocidal Gravid Ovitrap intervention impacts efficacy of *Aedes aegypti* control. J Appl Ecol. 2021;58: 2075–2086. doi:10.1111/1365-2664.13951

14. Harris AF, Sanchez Prats J, Nazario Maldonado N, Piovanetti Fiol C, García Pérez M, Ramírez‐Vera P, et al. An evaluation of *Bacillus thuringiensisisraelensis* (AM65</span> ‐52) treatment for the control of *Aedes aegypti* using vehicle‐mounted WALS application in a densely populated urban area of Puerto Rico. Pest Management Science. 2021;77: 1981–1989. doi:10.1002/ps.6227

15. Gato R, Menéndez Z, Prieto E, Argilés R, Rodríguez M, Baldoquín W, et al. Sterile insect technique: Successful suppression of an *Aedes aegypti* field population in Cuba. Insects. 2021;12: 469. doi:10.3390/insects12050469

16. Devine GJ, Vazquez-Prokopec GM, Bibiano-Marín W, Pavia-Ruz N, Che-Mendoza A, Medina-Barreiro A, et al. The entomological impact of passive metofluthrin emanators against indoor *Aedes aegypti*: A randomized field trial. PLOS Neglected Tropical Diseases. 2021;15: e0009036. doi:10.1371/journal.pntd.0009036

17. Holston J, Suazo-Laguna H, Harris E, Coloma J. DengueChat: A social and software platform for community-based arbovirus vector control. Am J Trop Med Hyg. 2021;105: 1521–1535. doi:10.4269/ajtmh.20-0808

18. Hustedt JC, Doum D, Keo V, Ly S, Sam B, Chan V, et al. Field efficacy of larvivorous fish and pyriproxyfen combined with community engagement on dengue vectors in cambodia: A randomized controlled trial. The American Journal of Tropical Medicine and Hygiene. 2021;105: 1265–1276. doi:10.4269/ajtmh.20-1088

19. Gopalan RB, Babu BV, Sugunan AP, Murali A, Ma MS, Balasubramanian R, et al. Community engagement to control dengue and other vector-borne diseases in Alappuzha municipality, Kerala, India. Pathog Glob Health. 115: 258–266. doi:10.1080/20477724.2021.1890886

20. Pinto R de A, Bauzer LGS da R, Borges DT, Lima JBP. Assessing the efficacy of two new formulations of larvicide pyriproxyfen for the control of *Aedes aegypti* using dissemination stations in two sites of Rio de Janeiro city. Mem Inst Oswaldo Cruz. 2020;115: e200271. doi:10.1590/0074-02760200271

21. Hamid NA, Alexander N, Suer R, Ahmed NW, Mudin RN, Omar T, et al. Targeted outdoor residual spraying, autodissemination devices and their combination against *Aedes* mosquitoes: field implementation in a Malaysian urban setting. Bull Entomol Res. 2020;110: 700–707. doi:10.1017/S0007485320000188

22. Newton-Sánchez OA, de la Cruz Ruiz M, Torres-Rojo Y, Ochoa-Diaz-López H, Delgado-Enciso I, Hernandez-Suarez CM, et al. Effect of an ecosystem-centered community participation programme on the incidence of dengue. A field randomized, controlled trial. Int J Public Health. 2020;65: 249–255. doi:10.1007/s00038-020-01345-y

23. Crawford JE, Clarke DW, Criswell V, Desnoyer M, Cornel D, Deegan B, et al. Efficient production of male *Wolbachia*-infected *Aedes aegypti* mosquitoes enables large-scale suppression of wild populations. Nature biotechnology. 2020;38: 482–492.

24. Gunathilaka N, Ranathunga T, Hettiarachchi D, Udayanga L, Abeyewickreme W. Field-based evaluation of novaluron EC10 insect growth regulator, a chitin synthesis inhibitor against dengue vector breeding in leaf axils of pineapple plantations in Gampaha District, Sri Lanka. Parasites & Vectors. 2020;13: 228. doi:10.1186/s13071-020-04109-y

25. Ahmad Zaki Z, Che Dom N, Ahmed Alhothily I. Efficacy of *Bacillus thuringiensis* treatment on *Aedes* population using different applications at high-rise buildings. Trop Med Infect Dis. 2020;5: 67. doi:10.3390/tropicalmed5020067

26. Bonnet E, Fournet F, Benmarhnia T, Ouedraogo S, Dabiré R, Ridde V. Impact of a community-based intervention on *Aedes aegypti* and its spatial distribution in Ouagadougou, Burkina Faso. Infectious Diseases of Poverty. 2020;9: 61. doi:10.1186/s40249-020-00675-6

27. Bohari R, Jin Hin C, Matusop A, Abdullah MR, Ney TG, Benjamin S, et al. Wide area spray of bacterial larvicide, *Bacillus thuringiensis israelensis* strain AM65-52, integrated in the national vector control program impacts dengue transmission in an urban township in Sibu district, Sarawak, Malaysia. PLOS ONE. 2020;15: e0230910. doi:10.1371/journal.pone.0230910

28. Garcia KKS, Versiani HS, Araújo TO, Conceição JPA, Obara MT, Ramalho WM, et al. Measuring mosquito control: adult-mosquito catches vs egg-trap data as endpoints of a cluster-randomized controlled trial of mosquito-disseminated pyriproxyfen. Parasites & Vectors. 2020;13: 352. doi:10.1186/s13071-020-04221-z

29. Lenhart A, Morrison AC, Paz-Soldan VA, Forshey BM, Cordova-Lopez JJ, Astete H, et al. The impact of insecticide treated curtains on dengue virus transmission: A cluster randomized trial in Iquitos, Peru. PLOS Neglected Tropical Diseases. 2020;14: e0008097. doi:10.1371/journal.pntd.0008097

30. Brelsfoard CL, Mains JW, Mulligan S, Cornel A, Holeman J, Kluh S, et al. *Aedes aegypti* males as vehicles for insecticide delivery. Insects. 2019;10: 230. doi:10.3390/insects10080230

31. Kittayapong P, Ninphanomchai S, Limohpasmanee W, Chansang C, Chansang U, Mongkalangoon P. Combined sterile insect technique and incompatible insect technique: The first proof-of-concept to suppress *Aedes aegypti* vector populations in semi-rural settings in Thailand. PLoS Negl Trop Dis. 2019;13: e0007771. doi:10.1371/journal.pntd.0007771

32. Barrera R, Amador M, Munoz J, Acevedo V. Integrated vector control of *Aedes aegypti* mosquitoes around target houses. Parasites & Vectors. 2018;11: 88. doi:10.1186/s13071-017-2596-4

33. Oo SZM, Thaung S, Maung YNM, Aye KM, Aung ZZ, Thu HM, et al. Effectiveness of a novel long-lasting pyriproxyfen larvicide (SumiLarv2MR) against *Aedes* mosquitoes in schools in Yangon, Myanmar. Parasites & Vectors. 2018;11: 16. doi:10.1186/s13071-017-2603-9

34. Ponlawat A, Harwood JF, Putnam JL, Nitatsukprasert C, Pongsiri A, Kijchalao U, et al. Field evaluation of indoor thermal fog and ultra-low volume applications for control of *Aedes aegypti* in Thailand. J Am Mosq Control Assoc. 2017;33: 116–127. doi:10.2987/16-6594.1

35. Abad-Franch F, Zamora-Perea E, Luz SLB. Mosquito-disseminated insecticide for Citywide vector control and Its potential to block arbovirus epidemics: Entomological observations and modeling results from amazonian Brazil. PLOS Medicine. 2017;14: e1002213. doi:10.1371/journal.pmed.1002213

36. Garziera L, Pedrosa MC, Souza FA, Gómez M, Moreira MB, Virginio JF, et al. Effect of interruption of over-flooding releases of transgenic mosquitoes over wild population of *Aedes aegypti*: two case studies in Brazil. Entomologia Experimentalis et Applicata. 2017;164: 327–339. doi:10.1111/eea.12618

37. Toledo ME, Vanlerberghe V, Rosales JP, Mirabal M, Cabrera P, Fonseca V, et al. The additional benefit of residual spraying and insecticide-treated curtains for dengue control over current best practice in Cuba: Evaluation of disease incidence in a cluster randomized trial in a low burden setting with intensive routine control. PLOS Neglected Tropical Diseases. 2017;11: e0006031. doi:10.1371/journal.pntd.0006031

38. Nagpal BN, Gupta SK, Shamim A, Vikram K, Srivastava A, Tuli NR, et al. Control of *Aedes aegypti* Breeding: A novel intervention for prevention and control of dengue in an endemic Zone of Delhi, India. PLOS ONE. 2016;11: e0166768. doi:10.1371/journal.pone.0166768

39. Setha T, Chantha N, Benjamin S, Socheat D. Bacterial Larvicide, *Bacillus thuringiensis israelensis* Strain AM 65-52 water dispersible granule formulation impacts both dengue vector, *Aedes aegypti* (L.) population density and disease transmission in Cambodia. PLoS Negl Trop Dis. 2016;10: e0004973. doi:10.1371/journal.pntd.0004973

40. Carvalho DO, McKemey AR, Garziera L, Lacroix R, Donnelly CA, Alphey L, et al. Suppression of a field population of *Aedes aegypti* in Brazil by sustained release of transgenic male mosquitoes. PLOS Neglected Tropical Diseases. 2015;9: e0003864. doi:10.1371/journal.pntd.0003864

41. Toledo ME, Vanlerberghe V, Lambert I, Montada D, Baly A, Stuyft PV der. No effect of insecticide treated curtain deployment on *Aedes* Infestation in a cluster randomized trial in a setting of low dengue transmission in Guantanamo, Cuba. PLOS ONE. 2015;10: e0119373. doi:10.1371/journal.pone.0119373

42. Quintero J, García-Betancourt T, Cortés S, García D, Alcalá L, González-Uribe C, et al. Effectiveness and feasibility of long-lasting insecticide-treated curtains and water container covers for dengue vector control in Colombia: a cluster randomised trial. Trans R Soc Trop Med Hyg. 2015;109: 116–125. doi:10.1093/trstmh/tru208

43. Mitchell-Foster K, Ayala EB, Breilh J, Spiegel J, Wilches AA, Leon TO, et al. Integrating participatory community mobilization processes to improve dengue prevention: an eco-bio-social scaling up of local success in Machala, Ecuador. Trans R Soc Trop Med Hyg. 2015;109: 126–133. doi:10.1093/trstmh/tru209

44. Barrera R, Amador M, Acevedo V, Caban B, Felix G, Mackay AJ. Use of the CDC autocidal gravid ovitrap to control and prevent outbreaks of *Aedes aegypti* (Diptera: Culicidae). J Med Entomol. 2014;51: 145–154. doi:10.1603/me13096

45. Tsunoda T, Kawada H, Huynh TTT, Le Luu L, Le SH, Tran HN, et al. Field trial on a novel control method for the dengue vector, *Aedes aegypti* by the systematic use of Olyset Net and pyriproxyfen in Southern Vietnam. Parasites & Vectors. 2013;6: 6. doi:10.1186/1756-3305-6-6

46. Lenhart A, Trongtokit Y, Alexander N, Apiwathnasorn C, Satimai W, Vanlerberghe V, et al. A cluster-randomized trial of insecticide-treated curtains for dengue vector control in Thailand. Am J Trop Med Hyg. 2013;88: 254–259. doi:10.4269/ajtmh.2012.12-0423

47. Castro M, Sánchez L, Pérez D, Carbonell N, Lefèvre P, Vanlerberghe V, et al. A community empowerment strategy embedded in a routine dengue vector control programme: a cluster randomised controlled trial. Trans R Soc Trop Med Hyg. 2012;106: 315–321. doi:10.1016/j.trstmh.2012.01.013

48. Arunachalam N, Tyagi BK, Samuel M, Krishnamoorthi R, Manavalan R, Tewari SC, et al. Community-based control of *Aedes aegypti* by adoption of eco-health methods in Chennai City, India. Pathog Glob Health. 2012;106: 488–496. doi:10.1179/2047773212Y.0000000056

49. Martínez-Ibarra JA, Nogueda-Torres B, Meda-Lara RM, Montañez-Valdez OD, Rocha-Chávez G. Combining two teaching techniques for young children on *Aedes aegypti* control: effects on entomological indices in western Mexico. Journal of Vector Ecology. 2012;37: 241–244. doi:10.1111/j.1948-7134.2012.00222.x

50. Abeyewickreme W, Wickremasinghe AR, Karunatilake K, Sommerfeld J, Kroeger A. Community mobilization and household level waste management for dengue vector control in Gampaha district of Sri Lanka; an intervention study. Pathog Glob Health. 2012;106: 479–487. doi:10.1179/2047773212Y.0000000060

51. Kittayapong P, Thongyuan S, Olanratmanee P, Aumchareoun W, Koyadun S, Kittayapong R, et al. Application of eco-friendly tools and eco-bio-social strategies to control dengue vectors in urban and peri-urban settings in Thailand. Pathog Glob Health. 2012;106: 446–454. doi:10.1179/2047773212Y.0000000059

52. Rizzo N, Gramajo R, Escobar MC, Arana B, Kroeger A, Manrique-Saide P, et al. Dengue vector management using insecticide treated materials and targeted interventions on productive breeding-sites in Guatemala. BMC Public Health. 2012;12: 931. doi:10.1186/1471-2458-12-931

53. Marcombe S, Darriet F, Tolosa M, Agnew P, Duchon S, Etienne M, et al. Pyrethroid resistance reduces the efficacy of space sprays for dengue control on the Island of Martinique (Caribbean). PLOS Neglected Tropical Diseases. 2011;5: e1202. doi:10.1371/journal.pntd.0001202

**S1 Text.**

**S1 Text.** A worked example of the Henderson formula used to estimate mosquito population suppression, along with an explanation of the quantile regression approach and the modeling strategy used to assess the impact of conflict of interest (COI) on reported outcomes.

**Table A in S1 Text.** Summary of conflict-of-interest (COI) reporting for the 51 included Aedes aegypti control trials published across 26 journals, including full journal names, abbreviations, COI-reporting fields, and study site. “Study site” indicates the country where the field trial was conducted (city/region noted when relevant). Journal abbreviations match those used in Table 1.

**Table B in S1 Text.** Summary of data extracted from studies evaluating Aedes aegypti population suppression methods. The table details the percentage reduction in mosquito populations and related entomological outcomes for various interventions**.**
