# Supplementary material for: Systematic review of Aedes aegypti control trials suggests publication bias related to author disclosure of conflicts of interest
Source: PLoS Negl Trop Dis. 2026 Jan 14;20(1):e0013914. doi: 10.1371/journal.pntd.0013914 (PMC12829951; doi:10.1371/journal.pntd.0013914)
Supplement: S2 Text — (PDF) [file pntd.0013914.s002.pdf]

S4 Code

R Code for Quantile Regression Analysis of Conflict of Interest Effects

```
#
=============================================================================
# CONFLICT OF INTEREST ANALYSIS - AEDES AEGYPTI SUPPRESSION
#
# This script runs quantile regression analyses to assess the relationship
# between conflict of interest (COI) declarations and reported efficacy
# in Aedes aegypti control studies.
#
# Two analyses are performed:
# 1. MAIN: All 226 comparisons from 51 studies
# 2. RESTRICTED: Only studies with explicit COI declarations
#
# Adjustments include:
# - Intervention type (Community, Insecticidal, Replacement, Suppression)
# - Geographic macro-regions (Africa, Americas, Asia, Oceania)
#
=============================================================================

library(readxl)
library(quantreg)
library(dplyr)
library(stringr)

rm(list = ls()); gc()

#
=============================================================================
# ANALYSIS 1: MAIN DATASET (ALL STUDIES)
#
=============================================================================

cat("\n=======================================================================
======\n")
cat("MAIN ANALYSIS: ALL STUDIES\n")
cat("=========================================================================
====\n\n")

# Read main dataset
wnv_main <- read_excel("S3_Table.xlsx", sheet = "MAIN")

# Rename columns
names(wnv_main) <- c("title", "coi", "intv", "r1", "reduction", "country")

# Clean variables
wnv_main$coi       <- factor(wnv_main$coi)
wnv_main$reduction <- as.numeric(wnv_main$reduction)
```

```
# Create macro-region groupings
wnv_main <- wnv_main %>%
  mutate(
    COUNTRY_clean = country %>%
      str_trim() %>%
      str_replace_all("\\.", "") %>%          # remove trailing dots
      str_replace("^Venezuel$", "Venezuela")  # fix misspelling
  ) %>%
  mutate(
    Region = case_when(
      COUNTRY_clean %in% c("USA", "Mexico") ~ "North America",
      COUNTRY_clean %in% c("Guatemala", "Nicaragua") ~ "Central America",
      COUNTRY_clean %in% c("Cuba", "Island of Martinique, Caribbean") ~
"Caribbean",
      COUNTRY_clean %in% c("Brazil", "Venezuela", "Peru", "Ecuador") ~ "South
America",
      COUNTRY_clean %in% c("Kenya") ~ "East Africa",
      COUNTRY_clean %in% c("Burkina Faso") ~ "West Africa",
      COUNTRY_clean %in% c("India", "Sri Lanka") ~ "South Asia",
      COUNTRY_clean %in% c("Cambodia", "Thailand", "Malaysia", "Vietnam",
"Myanmar") ~ "Southeast Asia",
      COUNTRY_clean %in% c("Australia") ~ "Oceania",
      TRUE ~ NA_character_
    ),
    MacroRegion = case_when(
      Region %in% c("North America", "Central America", "Caribbean", "South
America") ~ "Americas",
      Region %in% c("East Africa", "West Africa") ~ "Africa",
      Region %in% c("South Asia", "Southeast Asia") ~ "Asia",
      Region == "Oceania" ~ "Oceania",
      TRUE ~ NA_character_
    )
  )

# Set intervention reference level to "Insecticidal"
wnv_main$intv <- factor(
  wnv_main$intv,
  levels = c("Insecticidal", "Community", "Replacement", "Suppression")
)

# Summary statistics
cat("COI levels:", levels(wnv_main$coi), "\n")
cat("Intervention types:", levels(wnv_main$intv), "\n")
cat("MacroRegions:\n")
print(table(wnv_main$MacroRegion))
cat("\n")

cat("Summary of reduction values:\n")
print(summary(wnv_main$reduction))
cat("\n")

# Wilcoxon rank sum test
cat("--- Wilcoxon Rank Sum Test ---\n")
wilcox_main <- wilcox.test(reduction ~ coi, data = wnv_main)
print(wilcox_main)
```

```
cat("\n")

# Descriptive statistics by COI status
cat("--- Descriptive Statistics by COI Status ---\n")
means_main <- tapply(wnv_main$reduction, wnv_main$coi, mean, na.rm = TRUE)
sds_main <- tapply(wnv_main$reduction, wnv_main$coi, sd, na.rm = TRUE)
cat("Means:\n")
print(means_main)
cat("Standard Deviations:\n")
print(sds_main)
cat("\n")

# Quantile regression models
tau_mid <- 0.4999  # approximate median

# Model 1: FULL MODEL (COI + intervention + MacroRegion)
cat("=======================================================================\n"
)
cat("MODEL 1 (FULL): reduction ~ coi + intv + MacroRegion\n")
cat("=======================================================================\n"
)
fit1_main <- rq(reduction ~ coi + intv + MacroRegion, tau = tau_mid, data =
wnv_main)
sum1_main <- summary(fit1_main, se = "boot", R = 1000)
print(sum1_main$coefficients)
cat("\nCOI effect:\n")
print(sum1_main$coefficients[2, ])
cat("\n\n")

# Model 2: Reduced model (COI + intervention only)
cat("=======================================================================\n"
)
cat("MODEL 2: reduction ~ coi + intv\n")
cat("=======================================================================\n"
)
fit2_main <- rq(reduction ~ coi + intv, tau = tau_mid, data = wnv_main)
sum2_main <- summary(fit2_main, se = "boot", R = 1000)
print(sum2_main$coefficients)
cat("\nCOI effect:\n")
print(sum2_main$coefficients[2, ])
cat("\n\n")

# Model 3: Unadjusted
cat("=======================================================================\n"
)
cat("MODEL 3 (Unadjusted): reduction ~ coi\n")
cat("=======================================================================\n"
)
fit3_main <- rq(reduction ~ coi, tau = tau_mid, data = wnv_main)
sum3_main <- summary(fit3_main, se = "boot", R = 1000)
print(sum3_main$coefficients)
cat("\nCOI effect:\n")
print(sum3_main$coefficients[2, ])
cat("\n\n")
```

```
#
==============================================================================
# ANALYSIS 2: RESTRICTED DATASET (EXPLICIT COI DECLARATIONS ONLY)
#
==============================================================================

cat("\n========================================================================
======\n")
cat("RESTRICTED ANALYSIS: ONLY STUDIES WITH EXPLICIT COI DECLARATIONS\n")
cat("==========================================================================
====\n\n")

# Read restricted dataset
wnv_restricted <- read_excel("S3_Table.xlsx", sheet = "RESTRICTED")

# Rename columns
names(wnv_restricted) <- c("title", "coi", "intv", "r1", "reduction",
"country")

# Clean variables
wnv_restricted$coi       <- factor(wnv_restricted$coi)
wnv_restricted$reduction <- as.numeric(wnv_restricted$reduction)

# Create macro-region groupings
wnv_restricted <- wnv_restricted %>%
  mutate(
    COUNTRY_clean = country %>%
      str_trim() %>%
      str_replace_all("\\.", "") %>%
      str_replace("^Venezuel$", "Venezuela")
  ) %>%
  mutate(
    Region = case_when(
      COUNTRY_clean %in% c("USA", "Mexico") ~ "North America",
      COUNTRY_clean %in% c("Guatemala", "Nicaragua") ~ "Central America",
      COUNTRY_clean %in% c("Cuba", "Island of Martinique, Caribbean") ~
"Caribbean",
      COUNTRY_clean %in% c("Brazil", "Venezuela", "Peru", "Ecuador") ~ "South
America",
      COUNTRY_clean %in% c("Kenya") ~ "East Africa",
      COUNTRY_clean %in% c("Burkina Faso") ~ "West Africa",
      COUNTRY_clean %in% c("India", "Sri Lanka") ~ "South Asia",
      COUNTRY_clean %in% c("Cambodia", "Thailand", "Malaysia", "Vietnam",
"Myanmar") ~ "Southeast Asia",
      COUNTRY_clean %in% c("Australia") ~ "Oceania",
      TRUE ~ NA_character_
    ),
    MacroRegion = case_when(
      Region %in% c("North America", "Central America", "Caribbean", "South
America") ~ "Americas",
      Region %in% c("East Africa", "West Africa") ~ "Africa",
      Region %in% c("South Asia", "Southeast Asia") ~ "Asia",
      Region == "Oceania" ~ "Oceania",
      TRUE ~ NA_character_
    )
```

```
  )

# Set intervention reference level
wnv_restricted$intv <- factor(
  wnv_restricted$intv,
  levels = c("Insecticidal", "Community", "Replacement", "Suppression")
)

# Summary statistics
cat("COI levels:", levels(wnv_restricted$coi), "\n")
cat("Intervention types:", levels(wnv_restricted$intv), "\n")
cat("MacroRegions:\n")
print(table(wnv_restricted$MacroRegion))
cat("\n")

cat("Summary of reduction values:\n")
print(summary(wnv_restricted$reduction))
cat("\n")

# Wilcoxon rank sum test
cat("--- Wilcoxon Rank Sum Test ---\n")
wilcox_restricted <- wilcox.test(reduction ~ coi, data = wnv_restricted)
print(wilcox_restricted)
cat("\n")

# Descriptive statistics by COI status
cat("--- Descriptive Statistics by COI Status ---\n")
means_restricted <- tapply(wnv_restricted$reduction, wnv_restricted$coi, mean,
na.rm = TRUE)
sds_restricted <- tapply(wnv_restricted$reduction, wnv_restricted$coi, sd,
na.rm = TRUE)
cat("Means:\n")
print(means_restricted)
cat("Standard Deviations:\n")
print(sds_restricted)
cat("\n")

# Quantile regression models

# Model 1: FULL MODEL (COI + intervention + MacroRegion)
cat("======================================================================\n"
)
cat("MODEL 1 (FULL): reduction ~ coi + intv + MacroRegion\n")
cat("======================================================================\n"
)
fit1_restricted <- rq(reduction ~ coi + intv + MacroRegion, tau = tau_mid, data
= wnv_restricted)
sum1_restricted <- summary(fit1_restricted, se = "boot", R = 1000)
print(sum1_restricted$coefficients)
cat("\nCOI effect:\n")
print(sum1_restricted$coefficients[2, ])
cat("\n\n")

# Model 2: Reduced model
cat("======================================================================\n"
```

```
)
cat("MODEL 2: reduction ~ coi + intv\n")
cat("========================================================================\n"
)
fit2_restricted <- rq(reduction ~ coi + intv, tau = tau_mid, data =
wnv_restricted)
sum2_restricted <- summary(fit2_restricted, se = "boot", R = 1000)
print(sum2_restricted$coefficients)
cat("\nCOI effect:\n")
print(sum2_restricted$coefficients[2, ])
cat("\n\n")

# Model 3: Unadjusted
cat("========================================================================\n"
)
cat("MODEL 3 (Unadjusted): reduction ~ coi\n")
cat("========================================================================\n"
)
fit3_restricted <- rq(reduction ~ coi, tau = tau_mid, data = wnv_restricted)
sum3_restricted <- summary(fit3_restricted, se = "boot", R = 1000)
print(sum3_restricted$coefficients)
cat("\nCOI effect:\n")
print(sum3_restricted$coefficients[2, ])
cat("\n\n")

#
==============================================================================
# SUMMARY
#
==============================================================================

cat("\n========================================================================
======\n")
cat("ANALYSIS SUMMARY\n")
cat("========================================================================
====\n\n")

cat("MAIN ANALYSIS (All Studies):\n")
cat("  Wilcoxon p-value:", round(wilcox_main$p.value, 4), "\n")
cat("  Full Model COI effect (+ intervention + MacroRegion):\n")
cat("    β =", round(sum1_main$coefficients[2, "Value"], 3),
    ", SE =", round(sum1_main$coefficients[2, "Std. Error"], 3),
    ", p =", round(sum1_main$coefficients[2, "Pr(>|t|)"], 4), "\n\n")

cat("RESTRICTED ANALYSIS (Explicit COI Declarations Only):\n")
cat("  Wilcoxon p-value:", round(wilcox_restricted$p.value, 4), "\n")
cat("  Full Model COI effect (+ intervention + MacroRegion):\n")
cat("    β =", round(sum1_restricted$coefficients[2, "Value"], 3),
    ", SE =", round(sum1_restricted$coefficients[2, "Std. Error"], 3),
    ", p =", round(sum1_restricted$coefficients[2, "Pr(>|t|)"], 4), "\n\n")

cat("Analysis complete!\n")
```
